# Application of droplet digital PCR in minimal residual disease monitoring of rare fusion transcripts and mutations in haematological malignancies

**DOI:** 10.1038/s41598-024-57016-y

**Published:** 2024-03-16

**Authors:** Beca B. K. Ip, Anthony T. C. Wong, Janet Hei Yin Law, Chun Hang Au, Shing Yan Ma, James C. S. Chim, Raymond H. S. Liang, Anskar Y. H. Leung, Thomas S. K. Wan, Edmond S. K. Ma

**Affiliations:** 1 Division of Molecular Pathology, Department of Pathology, Hong Kong Sanatorium & Hospital, Happy Valley, Hong Kong; 2 Specialist in Haematology & Haematological Oncology, Causeway Bay, Hong Kong; 3 Department of Medicine and Comprehensive Oncology Centre, Hong Kong Sanatorium & Hospital, Happy Valley, Hong Kong; 4 Department of Medicine, The University of Hong Kong, Pok Fu Lam, Hong Kong

**Keywords:** Haematological cancer, Cancer genetics

## Abstract

Leukaemia of various subtypes are driven by distinct chromosomal rearrangement or genetic abnormalities. The leukaemogenic fusion transcripts or genetic mutations serve as molecular markers for minimal residual disease (MRD) monitoring. The current study evaluated the applicability of several droplet digital PCR assays for the detection of these targets at RNA and DNA levels (atypical *BCR::ABL1* e19a2, e23a2ins52, e13a2ins74, rare types of *CBFB::MYH11* (G and I), *PCM1::JAK2*, *KMT2A::ELL2, PICALM::MLLT10* fusion transcripts and CEBPA frame-shift and insertion/duplication mutations) with high sensitivity. The analytical performances were assessed by the limit of blanks, limit of detection, limit of quantification and linear regression. Our data demonstrated serial MRD monitoring for patients at molecular level could become “digitalized”, which was deemed important to guide clinicians in treatment decision for better patient care.

## Introduction

**Figure 1:**
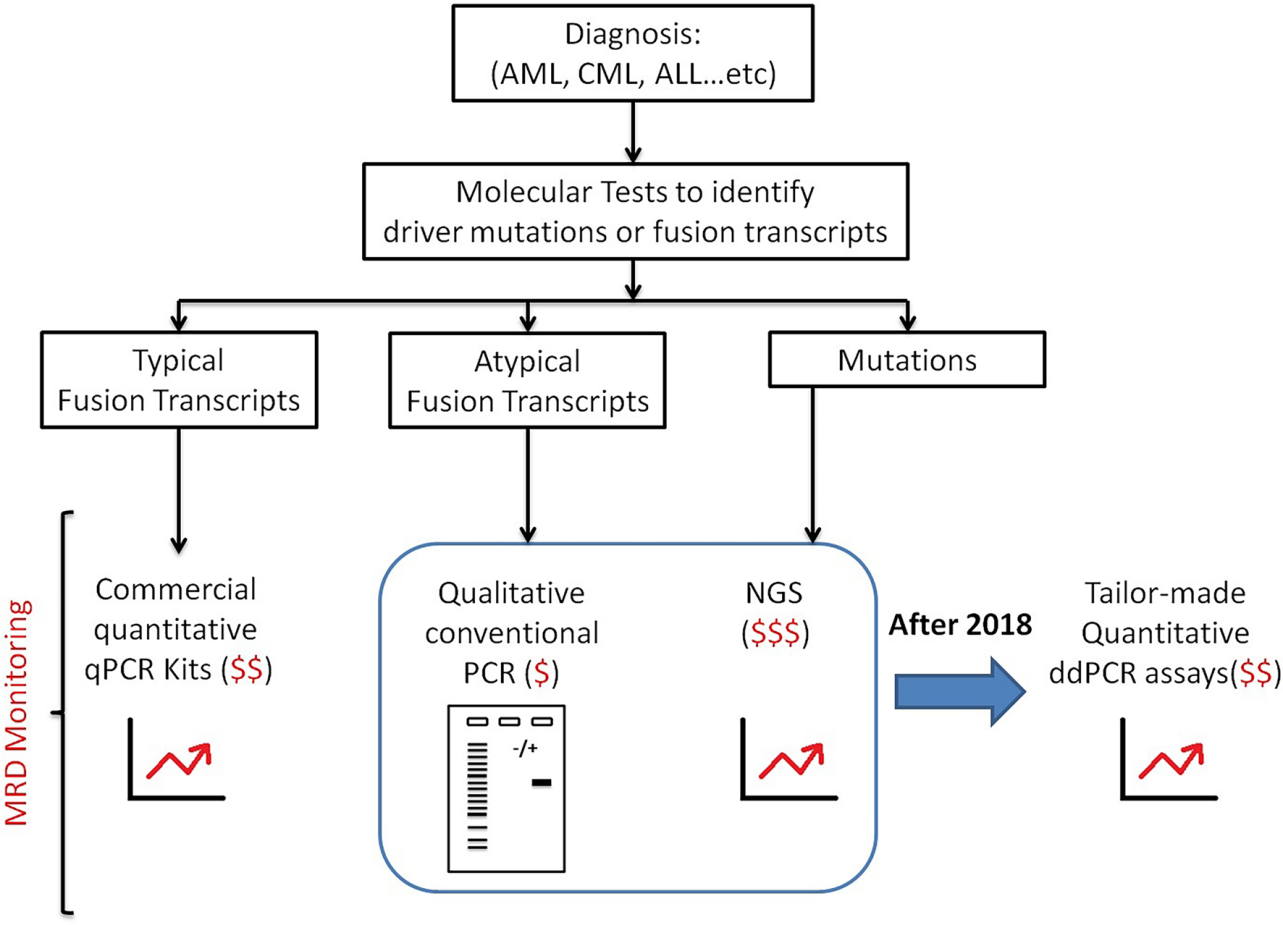
The rationale for the development of tailor-made ddPCR assays for MRD monitoring. Our laboratory aimed to provide a quantitative MRD monitoring scheme for the leukaemic patients carrying different types of driver mutations or atypical fusion transcripts at a price comparable to the commercially available qPCR assays. Of note, although lead time required to develop and optimize patient-specific ddPCR assays, the sensitivity and turnaround time would be improved after establishing the ddPCR assays.

Minimal residual disease (MRD) detection is essential for clinicians to evaluate the treatment response, risk stratification and prognostic prediction in patients with leukaemia. In acute lymphoblastic leukaemia (ALL) and acute myeloid leukaemia (AML), patients with positive MRD before and after allogeneic hematopoietic cell transplantation or at any time point during the course of treatment has a strong negative prognostic indication for relapse and worse overall survival^1–3^. In chronic myeloid leukaemia (CML), patients reaching a continual deep molecular response in a shorter time upon receiving tyrosine kinase inhibitor (TKI) treatment are predicted to have better outcome and eligible in considering the discontinuation of TKI therapy supported by the NCCN guidelines^4,5^. Various methods for MRD detection are available such as multi-parametric flow cytometry (FC), quantitative real-time (q)PCR and next generation sequencing (NGS). The cytometric and NGS methods, although available to detect a broad spectrum of targets for MRD monitoring, are less sensitive comparing to qPCR, and less applicable in CML. qPCR on the other hand detects specific target with higher sensitivity. Yet, when commercial kits are unavailable, in-house development of a well-validated qPCR assay is an expensive, laborious and lengthy procedure. It relies on the use of a standard curve for quantification and is prone to PCR inhibition. This potentially affects the sensitivity, reproducibility and accuracy of an assay when detecting targets at low level, which is crucial in measuring deep molecular response for MRD. In our laboratory before 2018, leukaemic patients carrying atypical fusion transcripts or driver mutations can only be monitored by conventional PCR or NGS at molecular level during their reassessments (Fig. 1). The former method although is cheap, it is a qualitative approach and the magnitude of changes in transcripts levels cannot be shown. On the other hand, while NGS is able to quantify the levels of mutant alleles in the form of variant allele frequencies (VAF), it is relatively expensive and time consuming. By partitioning samples into nanolitre droplets, an independent event of random distribution following the Poisson’s law, ddPCR greatly enhances the target abundance and enables absolution quantification via signal detection at the end point when droplets flow through the optical detector in single file. A number of ddPCR assays were developed with the aim to replace pre-existing qPCR assays for MRD monitoring with better sensitivity and precision^6–9^. In this study, we took a step forward to adapt this highly sensitive ddPCR system to detect rare leukaemic fusion transcripts and gene mutations. The aim was to design personalized MRD monitoring assays for patients, allowing clinicians to make prompt decision on treatment strategies upon detection of early signs of relapsing disease.

## Results

### ddPCR assays optimization

Primers and probes were designed in house following the recommendation by Bio-Rad Droplet Digital PCR Application Guide and the QXDx BCR-ABL %IS Kit protocol. Conventional PCR and Sanger sequencing was performed with all the diagnostic samples of the patients (all except *CBFB::MYH11* Type G) or gBLOCK Gene Fragments (*CBFB::MYH11* Type G) using the ddPCR primers for confirmation of fusion transcripts and mutations as well as to ensure the viability of the designed primer pairs (see Supplementary Table S3, S4 and S6 online). We have adopted the thermal cycling condition from the QXDx BCR-ABL %IS kit protocol and modified when necessary. In general, in addition to the enzyme activation, deactivation and droplet stabilization steps, a two-step PCR protocol was adopted in which annealing and extension of primers took place at the same temperature. A total of 40 amplification cycles including 5 cycles of a slightly lower annealing/extension temperature at 60 °C followed by 35 cycles at 64 °C would ensure good yield and specificity of the desired PCR amplicons (see Supplementary Table S5 online). The ddPCR assays for the three atypical *BCR::ABL1* fusion transcripts e19a2, e23a2ins52 and e13a2ins74 following this thermal cycling condition showed good separation of positive and negative clusters in both FAM and HEX channels (see Supplementary Fig. S1 online). The four clusters (double-negative, FAM-positive, HEX-positive and double positive) in the respective 2-D plots formed the typical rectangular shapes, indicating no cross reactivity between the FAM- and HEX-labeled probes. Similar phenomenon was observed when the same thermal cycling condition applied to the ddPCR assay for *PCM1*(exon29)::*JAK2*(exon11) (see Supplementary Fig. S2 online). However if we raised the primers annealing/extension temperature from 60 to 64 °C only for a total of 40 cycles, the negative clusters in both FAM and HEX channels became more compact. Furthermore, the signal intensity of FAM positive droplets increased from 5000 to 6000 units.

**Figure 2:**
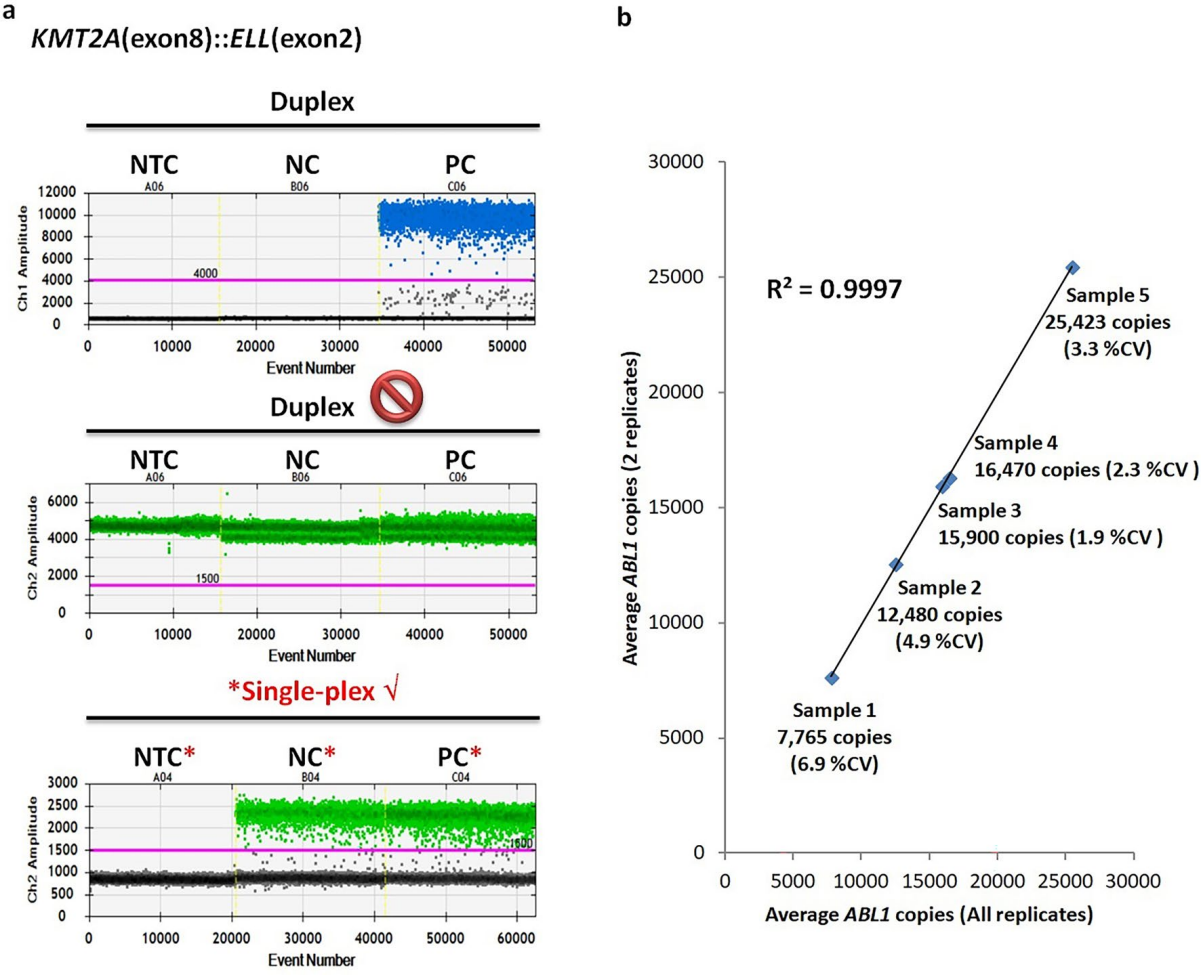
Optimization of *KMT2A*(exon 8)::*ELL*(exon 2) ddPCR assay. (**a**) 1-D plots of *KMT2A*(exon 8)::*ELL*(exon 2) ddPCR assay performed in duplex with *ABL1* causing the merging of positive and negative droplets in the HEX channel. Once the ddPCR assays for target and reference genes were performed in discrete wells (i.e. single-plex), the positive and negative droplets in the HEX channel were separated well. The target gene assay in the FAM channel was not affected even when performed in duplex. (NTC: no template control, NC: negative control, PC: positive control) (**b**) Regression analysis demonstrated the feasibility of extrapolating data of *ABL1* ddPCR assay from two wells to multiple wells of 4–10.

For *CBFB::MYH11* (Type G and I) (see Supplementary Fig. S3 online) and *KMT2A*(exon8)::*ELL*(exon2) (Fig. 2a) ddPCR assays, abnormally high signal intensities were detected in FAM- or HEX-negative clusters when both probes were present in the same reaction well, i.e. positive and negative clusters became indistingusible. Changing thermal cycling conditions did not help in such scenario. Simply setting up assays using FAM- and HEX-labeled probes in separate wells solved the issue for *CBFB::MYH11* (Type G) and *KMT2A*(exon8)::*ELL*(exon2). The primers for *CBFB::MYH11* Type I ddPCR assay adopted from Kadkol et al. 2004^10^ yielded an amplicon much larger than 200bp (the upper recommended amplicon size limit by the Bio-Rad ddPCR application guide). An additional 72 °C 2 min extension step was added and the annealing temperature was adjusted to 62 °C for 1.5 min with multiple attempts. Nevertheless, the disadvantage of this approach was exhaustion of patients’ samples when testing in replicates. In order to conserve the precious and limiting samples and determine the number of *ABL1* copies with respect to the number of wells used for detecting the fusion transcripts, we extrapolated the *ABL1* data from 2 wells by the following formula: Total *ABL1* copies = (*ABL1* copies in 2 wells ÷ 2) x number of wells for the detection of fusion transcripts. To prove the precision and consistency of the HEX-labeled probe in detecting *ABL1*, 5 samples were tested in 4–10 replicates to determine the *ABL1* copies in each well. Good amount of *ABL1* copies were detected in all 5 samples (7765–25,423 copies per well). Sample 4 and 5 showed good intra-run % coefficient of variation of 2.3 and 3.3%. Sample 1, 2 and 3 demonstrated good inter-run % coefficient of variation of 6.9, 4.9 and 1.9% respectively. Regression analysis demonstrated there was almost no difference in the *ABL1* copies when taking an average count of duplicates or all replicates, thus demonstrating the feasibility of extrapolating data from 2 wells to multiple wells (R^2^ = 0.9997) (Fig. 2b).

Gradient temperatures (68, 64, 60 and 58 °C) for the annealing/extension step were tested for the *PICALM*(exon19)::*MLLT10*(exon9) ddPCR assay. Positive and negative clusters in both FAM and HEX channels separated much further with lower annealing/extension temperature. 58 °C was preferable in comparison to 60 °C as the fluorescent signal intensity of FAM-positive droplets was higher (12,000 vs. 10,000 units), indicating higher PCR efficiency (see Supplementary Fig. S4 online).

**Figure 3:**
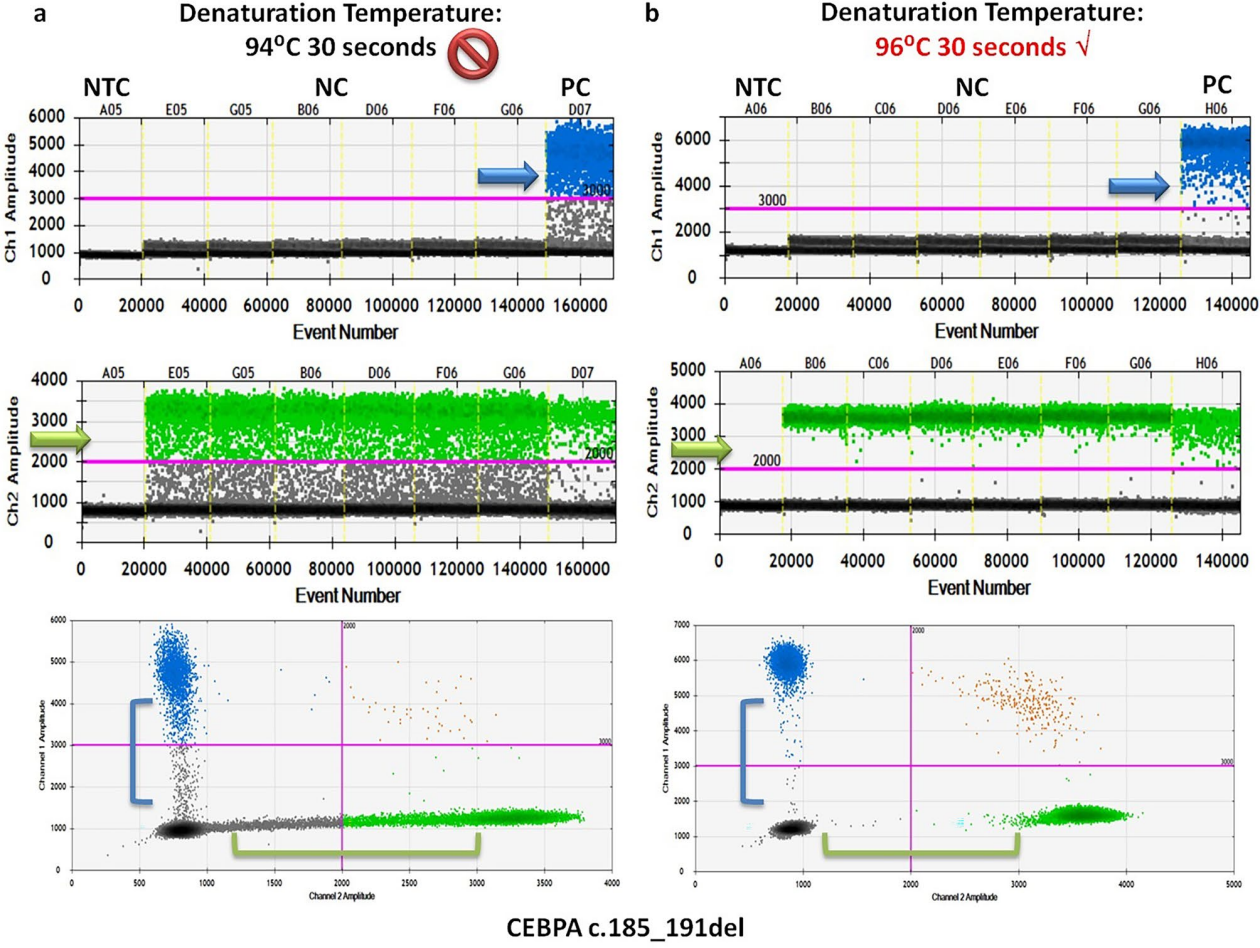
Optimization of CEBPA c.185_191del ddPCR assay. 1-D and 2-D plots of CEBPA c.185_191del ddPCR assay performed at 94 °C (**a**) and 96 °C (**b**) for the denaturation step. Heavy “raindrops” was observed with suboptimal denaturation temperature at 94 °C in both FAM- (blue arrow, **a**) and HEX-channels (green arrow, **a**). Higher denaturation temperature greatly enhanced the PCR efficiency as demonstrated by the loss of “raindrops” in the FAM- (blue arrow, **b**) and HEX- (green arrow, **b**) channels, and the positive clusters became more compact. (NTC: no template control, NC: negative control, PC: positive control).

For mutation detection ddPCR assays, an additional restriction enzyme digestion step using EcoRI-HF® (NEB, UK) was added to the general two-step PCR protocol with modifications when necessary. The ddPCR assay for CEBPA c.937_939dup was set up with minor adjustment by increasing the annealing/extension time from 1 to 1.5 min. Good separation of positive and negative clusters was achieved in both 1-D and 2-D plots (see Supplementary Fig. S5 online). Minor shifting of FAM-positive, HEX-positive and double-positive clusters was observed, forming a triangular instead of the typical rectangular shape. This was probably due to the interaction between the FAM-and HEX-labeled probes. Nevertheless, it did not affect the quantification of mutant and wild-type alleles and around 7000–10,000 total allele copies were detected per well during evaluation. When the general two-step PCR protocol with one annealing/extension temperature at 64 °C 1.5 min was applied to the CEBPA c.185_191del ddPCR assay, heavy “raindrops” was observed in both FAM- and HEX-channels (Fig. 3). The “raindrops” indicated suboptimal PCR efficiency. Additionally, the total copies per well were generally much lower than expected (around 2000–3000 vs. 7000–10,000 copies). Increasing the denaturation temperature from 94 to 96 °C improved the situation significantly, resulting in tightly formed FAM- and HEX-positive clusters in both 1-D and 2-D plots with little “raindrops”. The total copies per well were increased to 7000–10,000 copies during evaluation. Since CEBPA c.193_194del and c.185_191del ddPCR assays shared the same primer pair, identical thermal cycling condition was attempted initially and also applied to the CEBPA c.930_931insAAGCAG ddPCR assay as both were tailor-made for the same patient. The annealing/extension temperature at 64 °C 1.5 min seemed a little too stringent for the CEBPA c.930_931insAAGCAG ddPCR assay as the FAM-positive cluster remained rather close to the negative cluster (see Supplementary Fig. S6 online). By decreasing the annealing/extension temperature to 60 °C in both assays, the signal intensity of the FAM-positive droplets increased, separating further away from the negative clusters. The HEX-positive cluster appeared to be more compact in the CEBPA c.193_194del assay. Noting that the total copies per well were comparable in both conditions (7000–10,000 copies), a lower annealing/extension temperature seemed more desirable for better separation of positive and negative clusters in both assays.

### ddPCR analytical performance evaluation

**Figure 4:**
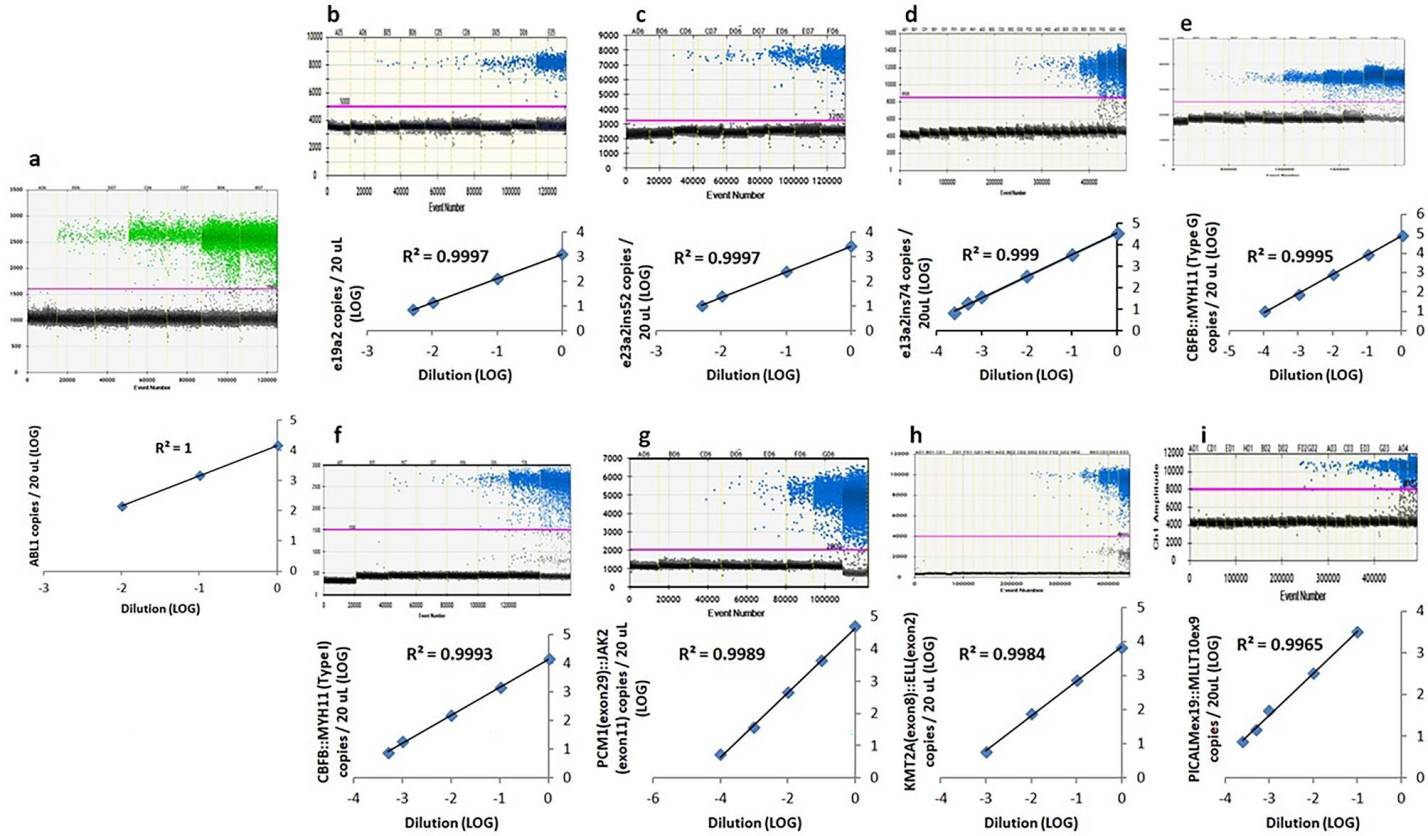
Analytical performance evaluation for *ABL1* (**a**), *BCR::ABL1* e19a2 (**b**), e23a2ins52 (**c**), e13a2ins74 (**d**), *CBFB::MYH11* Type G (**e**), Type I (**f**), *PCM1*(exon29)::*JAK2*(exon11) (**g**), *KMT2A*(exon8)::*ELL*(exon2) (**h**) and *PICALM*(exon19)::*MLLT10*(exon9) (**i**) ddPCR assays. Serial dilution of the diagnostic samples/gBLOCK gene fragment was performed to assess the linearity of each assay. The top panels showed the results in 1-D plots and the regression analyses gave R^2^ values ≥ 0.99 were demonstrated in the bottom panels.

**Figure 5:**
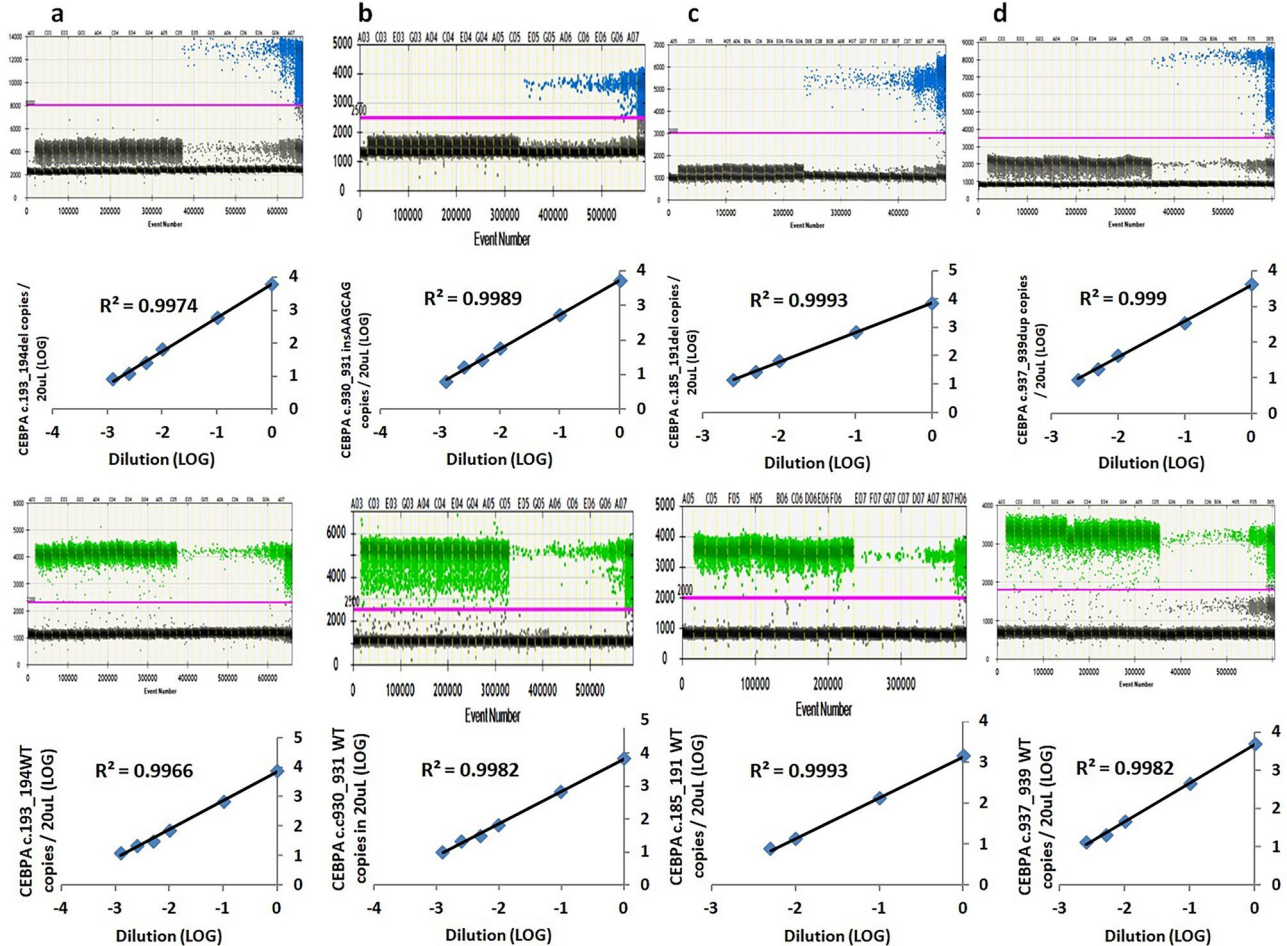
Analytical performance evaluation for CEBPA c.193_194del (**a**), c.930_931insAAGCAG (**b**), c.185_191del (**c**) and c.937_939dup (**d**) ddPCR assays. Serial dilution of the diagnostic samples was performed to assess the linearity of each assay (mutant alleles in the FAM-channel, wild-type alleles in the HEX-channel). The top (FAM-channel) and third (HEX channel) panels showed the results in 1-D plots and the regression analyses gave R^2^ values ≥ 0.99 were demonstrated in the second (FAM-channel) and bottom (HEX-channel) panels. (WT: wild-type).

The analytical performances of all assays were assessed by the limit of blank, limit of detection, limit of quantification as stated by Armbruster and Pry 2008^11^ with modified criteria and linearity. The limit of blank is defined by the highest number of positive droplets detected in a set of controls containing no target of interest (highest number of positive droplets + 3 standard deviation). The limit of blank was determined using 10–20 negative controls which were reverse-transcribed cDNA from archival RNA or DNA samples containing no fusion transcript or mutation of interest. No false positive droplet was detected in the FAM-channel, targeting the fusion transcripts or mutant alleles in all assays (i.e. limit of blank = 0 droplet/20 uL). The limit of detection was defined as the lowest number of droplets containing fusion transcript or mutant allele above the limit of blank. Thus a single positive droplet containing fusion transcript or mutant allele detected in a sample would be regarded as significant for all assays (i.e. limit of detection = 1 droplet/20 uL). Tenfold ± twofold serial dilutions were performed for each of the diagnostic samples (all except *CBFB::MYH11* Type G) or gBLOCK gene fragments (*CBFB::MYH11* Type G) to establish the limit of quantification and linearity. A pooled negative control cDNA of 1 ug and Buffer AE were used as diluent for fusion transcripts and mutation expression assays respectively. A total of 2–5 replicates were performed for the lowest point of the serial dilution. The limit of quantification was defined as the lowest copies of the established linear range with a minimum of 4 calibration points (except for the reference gene *ABL1*, with 3 calibration points only) (see Supplementary Table S1, S2 online, Figs. 4, 5). The limit of quantification (copies/20 uL) for most of the assays was down to single-digit copies in 20 uL, except for *BCR::ABL1* e23a2ins52, *CBFB::MYH11* Type G, CEBPA c.185_191del mutant assays which were ≤ 15 copies in 20 uL. The maximum copies in 20 uL hit within linear ranges were 14,610 for reference gene *ABL1*, and > 1400 for all the wild-type alleles of CEBPA. The R^2^ for all assays were ≥ 0.99 in linear regression analysis. For CEBPA mutation assays, the pre-defined variant allele frequencies (VAF) by NGS of all the diagnostic samples were at comparable level to the observed VAF by ddPCR (± 0.5LOG).

### Serial MRD Monitoring by ddPCR guides treatment strategy

Bone marrow aspirates or peripheral blood samples were collected from patients during their follow-up visits to the clinicians. ddPCR assays were performed to monitor their treatment responses at molecular level using a standard in-house protocol. A no template control, negative control and a pre-determined low positive control must be included in each run of ddPCR assay. The low positive controls contained the least amount of fusion transcripts or mutant alleles in the dilution series prepared from the diagnostic samples (all except *CBFB::MYH11* Type G) or gBLOCK gene fragment (*CBFB::MYH11* Type G). They served to validate each ddPCR run when the copies of fusion transcripts or mutant alleles in each 20 uL reaction were within ± 2 standard deviations. Results were expressed as the ratio in percentage, i.e. the copy number of the fusion transcript per copy of endogenous control gene (*ABL1*) or mutant allele per copy of the wild-type allele.

**Figure 6:**
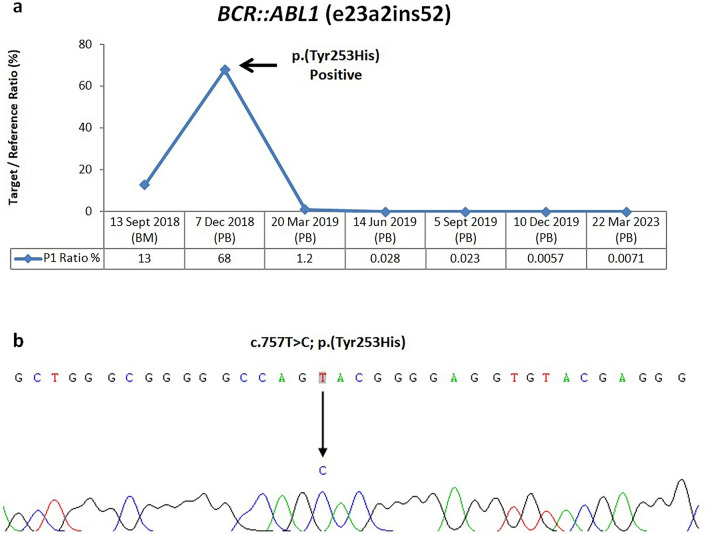
MRD monitoring for Patient 1. (**a**) Serial MRD monitoring for *BCR::ABL1* (e23a2ins52) by ddPCR. (BM: bone marrow, PB: peripheral blood) (**b**) Detection of *BCR::ABL1* kinase domain mutation by direct nucleotide sequencing. Nucleotide sequence showing a T to C substitution resulted in amino acid change p.(Tyr253His). This mutation was associated with acquired imatinib resistance causing a fivefold increase in transcript level (black arrow, **a**).

Patient 1 (P1) M/57Y was a chronic myeloid leukaemia (CML) patient with a novel in-frame *BCR::ABL1* fusion transcript, habouring a breakpoint in *BCR* exon 23 (first 33 bp), an insertion of a 52 bp *ABL1* pseudo-exon, to *ABL1* exon 2 (NM_004327.4:r.-452_3759::uguugggauuacaggcgugagccaccacgaccggucaaauugcugucuuaua::NM_005157.6:r.80_*1992) (Fig. 6). In September 2018, 13% of *BCR::ABL1* fusion transcript was detected in his first bone marrow aspirate collected after 9 months of first line tyrosine kinase inhibitor (TKI) imatinib treatment, indicating suboptimal response. The level raised by around fivefold to 68% in his peripheral blood after another three months of imatinib treatment, and a *BCR::ABL1* kinase domain mutation test was warranted. A point mutation causing an amino acid change in position 253 from Tyrosine to Histidine was identified, accounting for the resistance to imatinib treatment. Dasatinib was then used as second line TKI treatment, the level dropped below 10% three months later in his peripheral blood. Optimal response to TKI treatment was achieved as the level was maintained below 0.1% thereafter for 4 years according to the European LeukemiaNet (ELN) recommendations for the management of CML in 2013^12^.

Patient 2 (P2) F/67Y was an acute myeloid leukaemia (AML) patient with *BCR::ABL1* (e19a2) fusion transcript (see Supplementary Fig. S7 online). Before 2019, her disease monitoring was based on qualitative method using conventional PCR with a sensitivity of around 10^–3^ to 10^–4^^13,14^. With the tailor-made ddPCR assay, quantification of the fusion transcript level at each reassessment time point was achievable with greater sensitivity of around 10^–4.5^ to 10^–5^. She has maintained a stable disease with the level < 0.01% in all her peripheral and marrow blood samples.

Patient 3 (P3) F/34Y was chronic myeloid leukaemia patient who presented in B-lymphoblastic crisis with another novel in-frame *BCR::ABL1* fusion transcript (see Supplementary Fig. S7 and S10 online). The breakpoint resulted in a truncated e13a2 and a 74bp insertion of a non-templated A and the *ABL1* pseudo-exon (NM_004327.4:r.-452_2684::aacccaccuuggccucccaaaguguugggauuacaggcgugagccaccacgaccggucaaauugcugucuuaua::NM_005157.6:r.80_*1992). Her diagnostic bone marrow aspirate collected in February 2023 contained high level of fusion transcript (153%), concordant with a strongly positive single conventional PCR result using published primer sets^13^ alongside with the commercial positive control of *BCR::ABL1* p210 b2a2. Within a month of treatment by hyper-CVAD chemotherapy and dasatininb, the level dropped to 7.2% in her bone marrow, concordant with a positive single conventional PCR result. Multi-parametric flow cytometry of bone marrow cells also showed detectable MRD result of 0.17%. Monthly MRD monitoring by ddPCR in her bone marrow samples showed a decreasing trend to < 0.1%, concordant with the weakly positive nested conventional PCR results, albeit flow cytometry gave undetectable MRD results but at a lower analytical sensitivity validated to 0.1%. When the level in her bone marrow collected in May 2023 dropped below 0.01% by ddPCR, it became undetectable in the nested conventional PCR. Two months later, it was undetectable in her bone marrow both by ddPCR with a 10^–5^ sensitivity and flow cytometry. The patient received a matched unrelated hematopoietic stem cell transplantation (HSCT) and remained well.

Patient 4 (P4) F/58Y and 5 (P5) F/66Y were acute myeloid leukaemia (AML) patients with rare *CBFB::MYH11* Type G and I fusion transcripts respectively (see Supplementary Fig. S8 online). P4 was pretreated elsewhere outside Hong Kong and hence the fusion transcript was at low level in her first bone marrow sample (0.0052%) in April 2018. This notwithstanding, the breakpoint in *CBFB::MYH11* was identifiable. It remained detectable at low level (> 0.001%) in her reassessment marrow and peripheral blood samples with a decreasing trend till February 2019. It became undetectable for over a year until December 2020 with 10^–4.5^–10^–5^ sensitivity. The sudden change of MRD status from negative to positive, though remained at very low level (< 0.001%), advocated a closer monitoring regime. Monthly MRD monitoring by ddPCR showed an increased level > 0.001% in her bone marrow collected in January 2021. It gradually decreased < 0.001% and became undetectable in late March 2021 with 10^–4.5^–10^–5^ sensitivity. She maintained a stable disease with a negative MRD status for 2 years.

The diagnostic bone marrow aspirate collected in April 2019 of P5 contained high level of Type I fusion transcript (158%). After 5 months of treatment (7:3 induction followed by high dose cytosine arabinoside (HDAC) consolidation), the level dropped < 0.1%. She maintained a stable disease at low level (< 0.01%) or undetectable with 10^–4.5^ to 10^–5^ sensitivity for 4 years.

Patient 6 (P6) M/40Y was an acute myeloid leukaemia (AML) patient with *PICALM::MLLT10* fusion transcript habouring a breakpoint in *PICALM* exon 19 and *MLLT10* exon 9 (NM_007166.4: r.-304_1944::NM_001195626.3:r.700_*1647) (see Supplementary Fig. S8 online). His diagnostic bone marrow aspirate collected in early 2022 contained high level of fusion transcript (84%). Although the level dropped, it failed to reach below 0.1% in his bone marrow even after more than 5 months of treatment (7:3 induction followed by HDAC consolidation) and increasing trend of MRD heralded an extramedullary relapse that was managed by salvage chemotherapy. The patient received an allo-HSCT from matched unrelated donor (MUD) in January 2023 and day 22 MRD monitoring by ddPCR showed optimal response with level < 0.01%, and became undetectable 4 months later with 10^–4.5^ sensitivity. Unfortunately, in August 2023, he turned MRD + and increased by 51-fold to 0.45% by October 2023. The patient was managed by withdrawal of immunosuppressive therapy but donor lymphocyte infusion (DLI) was not an option since the MUD was from an overseas donor.

**Figure 7:**
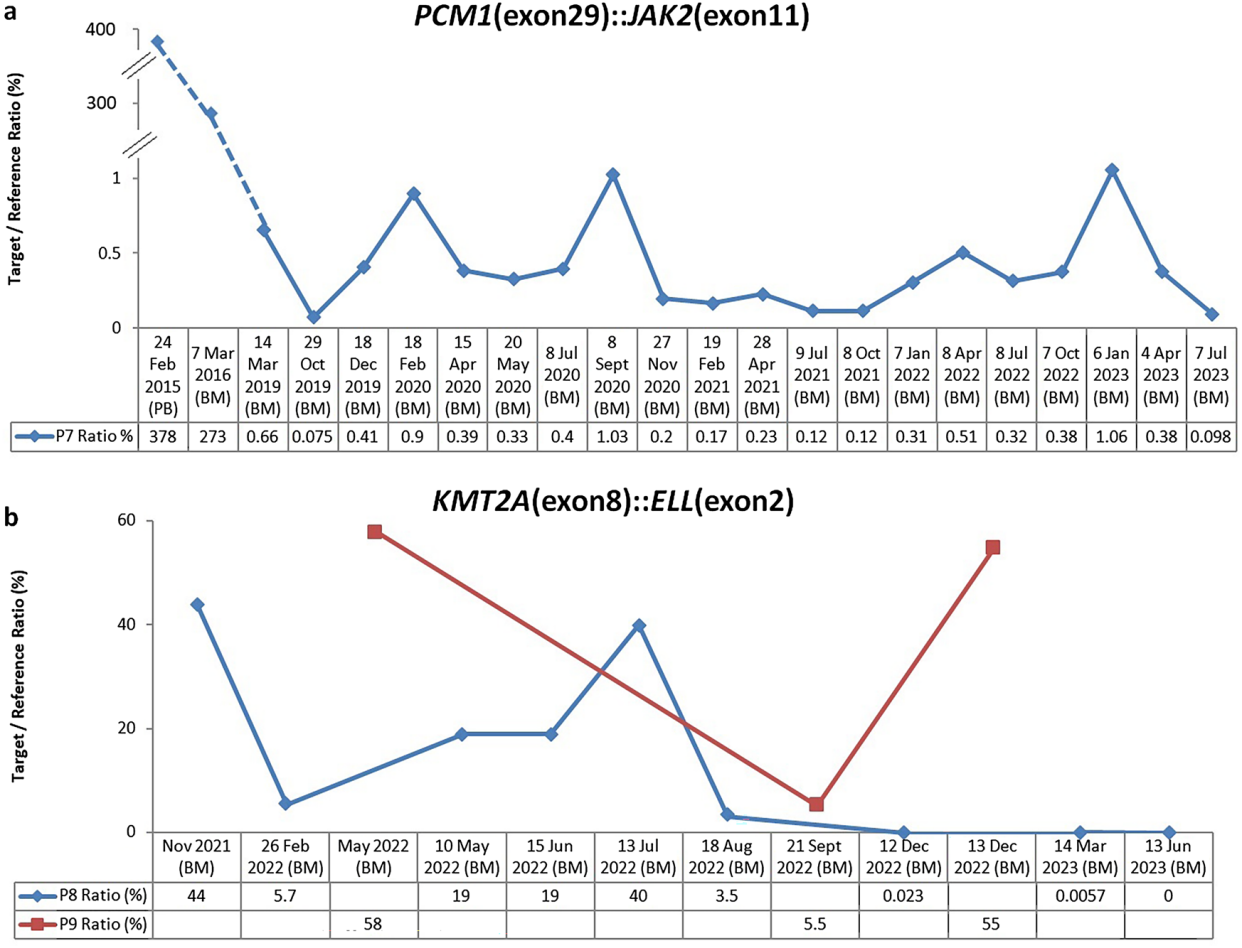
MRD monitoring for Patient 7, 8 and 9. Serial MRD monitoring for *PCM1*(exon29)*::JAK2*(exon11) (**a**) and *KMT2A*(exon8)::*ELL*(exon2) (**b**) by ddPCR. (BM: bone marrow, PB: peripheral blood).

Patient 7 (P7) M/73Y was a chronic eosinophilic leukaemia (CEL) patient who was detected to have a *PCM1::JAK2* fusion transcript that showed a breakpoint in *PCM1* exon 29 and *JAK2* exon 11 (NM_006197.3:r.-422_4827::NM_004972.3:r.1327_*1392) (Fig. 7a). The fusion transcript and its breakpoint were initially identified in his bone marrow aspirate collected in March 2016 which contained high level of fusion transcript (273%). Archival RNA of this patient which was extracted from his peripheral blood collected in February 2015 contained even higher level (378%). He was given a Janus kinase inhibitor (Ruxolitinib) in April 2016. Unfortunately, in February 2019 there was disease transformation to B-acute lymphoblastic leukaemia. The patient completed hyperCVAD and received a haploidentical HSCT from his daughter. Serial monitoring by ddPCR since October 2019 showed persistently detectable and fluctuating MRD levels. In the 2-year interval from October 2019 to October 2022, the patient received DLI for 6 doses. Two more doses of DLI were administered in April and May 2023 for increased MRD levels. The patient was now 50 months post-HSCT and remained in MRD + complete remission.

Patient 8 (P8) F/41Y and 9 (P9) F/60Y were acute myeloid leukaemia (AML) patients with *KMT2A::ELL* fusion transcript habouring a breakpoint in *KMT2A* exon 8 and *ELL* exon 2 (NM_005933.3: r.-23_4086::NM_006532.3:r.136_*2089) (Fig. 7b). The diagnostic bone marrow aspirate of P8 collected in November 2021 contained high level of fusion transcript (44%), and decreased to 5.7% in February 2022 following 7:3 induction chemotherapy. However, she relapsed in May 2022 and the level increased continually to 40% in July 2022. After re-induction therapy by FLAG-idarubicin and venetoclax, it gradually decreased to < 0.01% in March 2023, and became undetectable with 10^–4.5^ sensitivity in June 2023. The patient received a haploidentical HSCT from a cousin.

The diagnostic bone marrow aspirate of P9 collected in May 2022 contained high level of fusion transcript (58%). It declined to 5.5% in September 2022 after 7:3 induction followed by HDAC consolidation treatment. However, she relapsed 3 months later at a level comparable to her diagnostic sample even with a more aggressive fludarabine-based salvage therapy. The patient was planned for haplo-HSCT from daughter but was deemed not fit due to multiple co-morbidities.

Patients 10–14 (P10 M/62Y, P11 M/39Y, P12 F/46Y, P13 F/49Y and P14 F/43Y) are acute myeloid leukaemia (AML) patients habouring mono- or bi-allelic CEBPA (NM_004364.5) mutations (see Supplementary Fig. S9 online). The diagnostic bone marrows collected from patients P10–P13 contained high levels CEBPA mutant alleles with a mutant to wild-type allele ratio 75% or above. After treatment, the level of mutant alleles in all patients were detected at very low level (mostly < 0.01%) or undetectable with sensitivity of 10^–4.5^ to 10^–5^.

## Discussion

**Figure 8:**
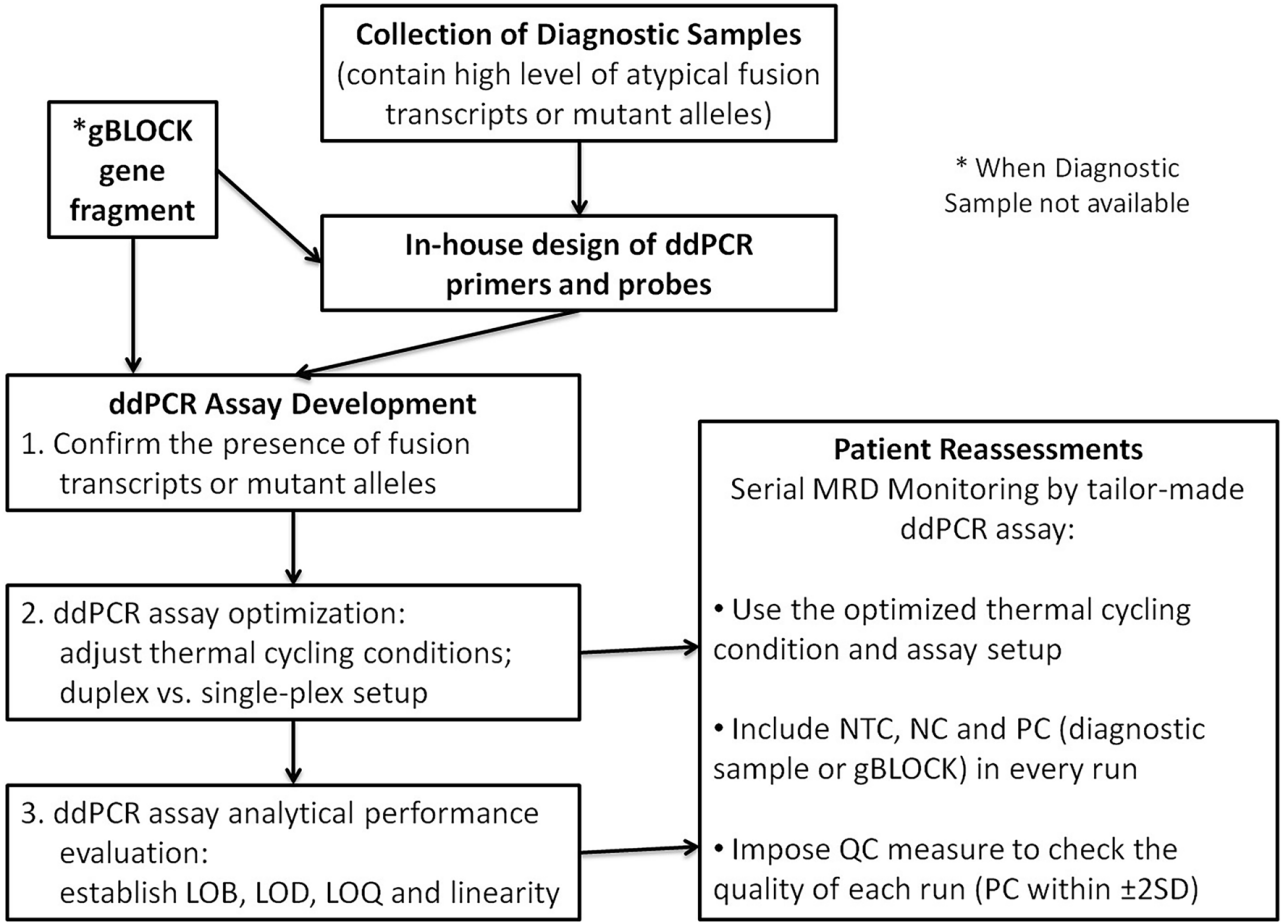
Guidelines for best practice in designing a tailor-made ddPCR assay for MRD monitoring. The diagnostic samples of patients are important for ddPCR assay development and serve as positive controls in every reassessment assay. (LOB: limit of blank, LOD: limit of detection, LOQ: limit of quantification, NTC: no template control, NC: negative control, PC: positive control, QC: quality control, SD: standard deviation).

Our study demonstrated the feasibility of applying ddPCR platform in MRD monitoring for leukaemia patients with high sensitivity. It is noteworthy that the diagnostic samples are important for the assay development. The idea of the best practice in developing a personalized ddPCR assay for MRD monitoring is summarized in Fig. 8. Prior to analytical performance evaluation, the diagnostic samples were used for tuning thermal cycling conditions of the ddPCR assays. Technically challenging assays including the *CBFB::MYH11* Type G and I, *KMT2A::ELL* and some of the CEBPA mutant alleles required multiple attempts in optimization. The assays for detecting the three listed fusion transcripts were set up separately from the *ABL1* reference gene assay instead of within the same well (i.e. single-plex). This was due to inseparable positive and negative clusters or the abnormal increase of background signal in the negative control wells to a comparable level of the positive controls either in the FAM or HEX channel when they are coupled within the same well. GC-rich targets might form unknown secondary structures, hindering the extension of primers or binding of probes. For the *CBFB:MYH11* Type I and some of the CEBPA mutation assays, an additional 72 °C extension step or increasing the denaturation temperature to 96 °C helped solving the issue and increased the distance between positive and negative clusters, reduced the heavy “raindrops” that severely affecting the quantification of copy numbers. The NGS VAF of the diagnostic samples served as another checkpoint for the accuracy of ddPCR assays, which cannot be accomplished by the synthetic gene fragments. While extensive evaluation work with more controls, replicates or inter-run comparisons were desirable, we had to balance between the reagent costs, exhaustion of archival or diagnostic samples and turnaround time.

Since the establishment of ddPCR platform in our laboratory, there was a shift of paradigm in MRD testing requested by the clinicians in our laboratory. Without the ddPCR platform and commercial qPCR assays, the MRD monitoring of CML patient habouring *BCR::ABL1* e19a2 was only achievable by a qualitative conventional PCR with a sensitivity of around 10^–3^ to 10^–4^^13,14^. The patient was MRD negative throughout the treatment course, and the minor changes of MRD status from negative to positive only became detectable by ddPCR with an improved sensitivity of around 10^–4.5^ to 10^–5^. A nested conventional PCR for MRD testing, though might enhance the sensitivity, it is only a semi quantitative assay when running alongside with the diagnostic sample, limiting its predictive value of treatment responsiveness.

ddPCR visualized the intensities of gel bands into copies of target as in the case of *BCR::ABL1* e13a2ins74. Although the results obtained by ddPCR did not involve the use of a calibrator to align with the International Scale, it revealed an equivalence of major molecular response (MMR) and MR4 after only 2 and 3 months and of treatment respectively. It is vital for clinicians to identify the milestone in terms of molecular response status that their patients have achieved throughout the treatment course. A sustainable deep molecular response determined by accurate and sensitive methods such as ddPCR might predict a treatment-free remission even after cessation of therapy^15,16^.

AML is a heterogeneous haematological disorder with a great variety of chromosomal and genetic abnormalities bearing prognostic values. In addition to the most widely used methods such as flow cytometry and qPCR, NGS and ddPCR are the two promising techniques for MRD monitoring with 10^–2^ to 10^–4^ sensitivity as recommended by the European LeukemiaNet (ELN) in 2022^17^. Patients carrying fusion transcripts such as *CBFB::MYH11*, *KMT2A::MLLT3* and mutated genes such as NPM1 are well-suited to be monitored by qPCR^18–20^. In our laboratory, we have applied our myeloid genes NGS panel^21^ for monitoring gene mutations in AML patients before the establishment of ddPCR platform. Despite a relatively long turnaround time and expensive cost, it offered a quantitative measure and particularly useful in comprehensively detecting actionable gene mutations such as FLT3, IDH1, IDH2 and NPM1 as their corresponding targeted therapies became available or in on-going clinical trials^17,22–26^.

ddPCR served as an alternative approach with shorter turnaround time at a cheaper cost. Various ddPCR assays detecting these actionable gene mutations have been successfully developed by other groups^27–29^ in which further exploitation of these assays in MRD monitoring is feasible. Other NGS platforms such as the RNA-based fusions panel^30^ and the DNA-based long read sequencing^31^, though hold great potentials in identifying cryptic and novel gene rearrangements at diagnosis, are not cost- and time-effective methods for MRD monitoring due to limited sensitivity. Potentially actionable targets involving gene rearrangements such as *KMT2A* with ongoing clinical trials^26,32^ warrant sensitive and accurate quantification platform to measure the treatment responses. The RNA-based fusion ddPCR assays recently developed by our group, in addition to previous^31^ and current studies and by others^33^, provide a relatively simple and economical approach to track the changes of levels in these disease-driving gene fusions, allowing therapeutic intervention promptly. Of note, ELN recommendation in 2018 and 2021 suggested all mutated genes detected by NGS panels as potential MRD markers except those related to clonal haematopoiesis^19,20^. Nevertheless, a recent publication demonstrated the usefulness of ddPCR in monitoring these mutated genes in a subset of non-relapsing, post-transplanted AML-patients^34^.

In sum, this profoundly encouraged further application of ddPCR in monitoring any gene mutations beyond actionable ones that were identified by NGS panels. The framework of developing DNA-based ddPCR assays such as CEBPA in our study can be implemented on other mutated genes for prediction of disease progression and evaluation of treatment responses.

In conclusion, the personalized ddPCR assays presented in this study kept patients under close surveillance at molecular level during the course of treatment. The importance of this application was highlighted by the Patient 1 carrying an actionable, yet atypical *BCR::ABL1* fusion transcript (e23a2ins52), in maintaining a deep molecular response for 4 years after a relapse and timely switch to second line treatment. The ddPCR assay also helped to guide DLI therapy in Patient 7 over the course of more than 2 years.

## Methods

### Patient samples and control materials

Bone marrow or peripheral blood samples were obtained from individual patients at diagnosis and follow-ups. RNA or DNA was extracted from each sample using QIAamp RNA Blood Mini Kit and QIAamp DNA Blood Mini Kit respectively (Qiagen, Germany). Both extracted RNA and DNA was quantified using Nanodrop 2000c Spectrophotometer (Thermo Fisher Scientific USA) and qualified by the OD A260/280 ratio. The negative controls used in analytical performance evaluation and reassessment assays were pre-existing de-identified archival RNA and DNA samples from individuals with no disease indication of the respective haematological malignancy. The positive controls used in the analytical performance evaluation and reassessment assays were the diagnostic samples of the patients. The presence of fusion transcripts and mutant alleles in the diagnostic samples were previously identified by NGS or Sanger sequencing in our or the referral laboratories. However when they were unavailable, artificial gBLOCK gene fragments (Integrated DNA Technologies, USA) containing the fusion transcripts or mutations of interest were synthesized as an alternative. Conventional PCR and Sanger sequencing was performed on the positive controls (diagnostic samples and gBLOCK gene fragments) and some reassessment samples for confirmation of fusion transcripts and mutant alleles as well as the detection of resistance mutation. Commercial positive control of *BCR::ABL1* p210 b2a2 (invivoscribe, USA) RNA was also used for MRD monitoring of *BCR::ABL1* (e13a2ins74) by conventional PCR.

The study was performed in accordance with the Declaration of Helsinki. Written informed consent was obtained from all attending clinicians of the listed patients in this retrospective study. The study was approved by the HKSH Medical Group Research Committee (RC Ref. No: RC-2023-13).

### Minimal residual disease monitoring on RNA level by ddPCR

For each sample, 1 ug RNA was reverse transcribed to 20 uL complementary DNA (cDNA) using random primers of the iScript Select cDNA Synthesis Kit (Bio-Rad, USA). In general, ddPCR reactions were prepared by adding 6.4 uL cDNA into 1 × ddPCR Supermix for Probes (No dUTP) on Bio-Rad QX200 Droplet Digital PCR System (Bio-Rad, USA). Each 20 uL reaction contained a 900 nM target-specific primer pair and a 250 nM FAM-labeled target specific probe (see Supplementary Table S3, S4 and S6 online). All assays were set up as duplex or single-plex with the reference gene *ABL1* using the 250 nM *ABL1*-specific primer pair and the 250 nM HEX-labeled *ABL1*-specific probe (see Supplementary Table S3, S4 and S6 online). Thermal cycling was performed using C1000 Touch Thermal Cycler (Bio-Rad, USA) at ramp rate 2 °C per second with various conditions (see Supplementary Table S5 and S6 online).

### Minimal residual disease monitoring on DNA level by ddPCR

ddPCR reactions were prepared using 1 × ddPCR Supermix for Probes (No dUTP) (Bio-Rad, USA) with 5U EcoRI-HF® (NEB, UK) on Bio-Rad QX200 Droplet Digital PCR System (Bio-Rad, USA). In general, 40 ng DNA was used for each 20 uL reaction, containing a 900 nM region-specific primer pair, 250 nM FAM-labeled probe and 250 nM HEX-labeled probe (see Supplementary Table S3, S4 and S6 online). Thermal cycling was performed using C1000 Touch Thermal Cycler (Bio-Rad, USA) at ramp rate 2 °C per second with various conditions (see Supplementary Table S5 and S6 online).

### ddPCR data acquisition and analysis

Raw data acquisition and analysis were performed using QuantaSoft version 1.7.4.0917 (Bio-Rad, USA). Data collected from various PCR replicates per cDNA or DNA sample were manually merged for calculation of total copies. For minimal residual disease monitoring on RNA level, the fusion transcript expression level was normalized to the reference gene *ABL1*, and calculated as (copies of fusion transcript ÷ copies of *ABL1*) × 100%. For minimal residual disease monitoring on DNA level, the allelic ratio was calculated as (copies of mutant allele ÷ copies of wild-type allele) × 100%.

### Supplementary Information

Supplementary Information.

## Data Availability

The datasets generated during and/or analyzed during the current study are available from the corresponding author on reasonable request.
