# Supplementary material for: Application of droplet digital PCR in minimal residual disease monitoring of rare fusion transcripts and mutations in haematological malignancies
Source: Sci Rep. 2024 Mar 16;14:6400. doi: 10.1038/s41598-024-57016-y (PMC10944481; doi:10.1038/s41598-024-57016-y)
Supplement: Supplementary file 1 — Supplementary Information. [file 41598_2024_57016_MOESM1_ESM.docx]

Application of droplet digital PCR in minimal residual disease monitoring of rare fusion transcripts and mutations in haematological malignancies

Authors

Beca B K Ip^1^, Anthony T C Wong^1^, Janet Hei Yin Law^1^, Chun Hang Au^1^, Shing Yan Ma^2^, James C S Chim^3^, Raymond H S Liang^3^, Anskar Y H Leung^4^, Thomas S K Wan^1^, Edmond S K Ma^1*^

^1^Division of Molecular Pathology, Department of Pathology, Hong Kong Sanatorium & Hospital, Hong Kong

^2^Specialist in Haematology & Haematological Oncology, Hong Kong

^3^Department of Medicine and Comprehensive Oncology Centre, Hong Kong Sanatorium & Hospital, Hong Kong

^4^Department of Medicine, The University of Hong Kong, Hong Kong

*corresponding author, Email: eskma@hksh.com

Supplementary Information


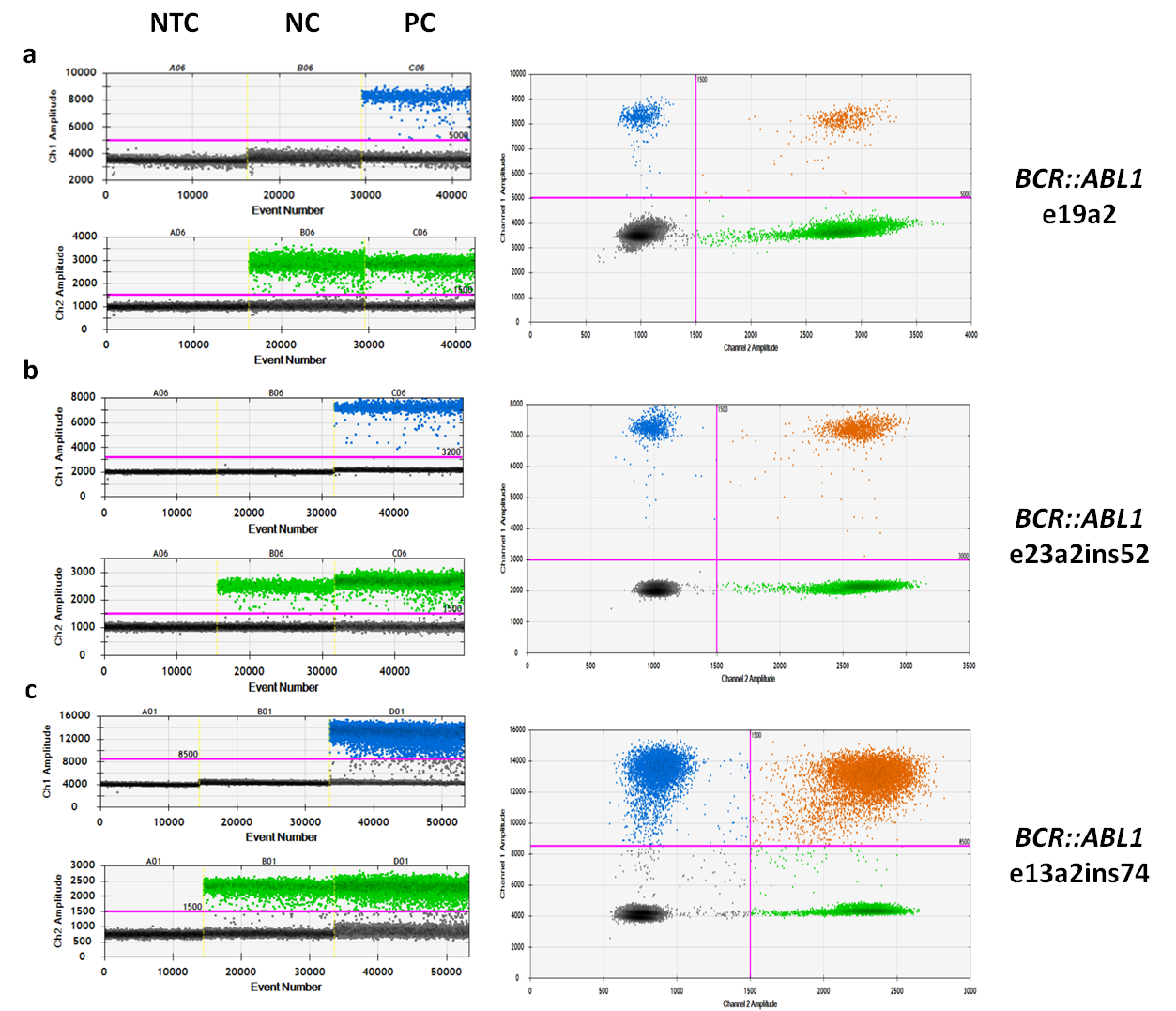


**Supplementary Figure S1**: Optimization of atypical *BCR::ABL1* ddPCR assays. 1-D and 2-D plots of atypical *BCR::ABL1* fusion transcripts (a) e19a2, (b) a breakpoint in *BCR* exon 23 (first 33 bp), with an insertion of a 52 bp *ABL1* pseudo-exon, to *ABL1* exon 2 (e23a2ins52) and (c) a truncated e13a2 with a 74bp insertion of a non-templated A and the *ABL1* pseudo-exon (e13a2ins74) ddPCR assays. Good separation of positive and negative clusters in both channels. (NTC: no template control, NC: negative control, PC: positive control)


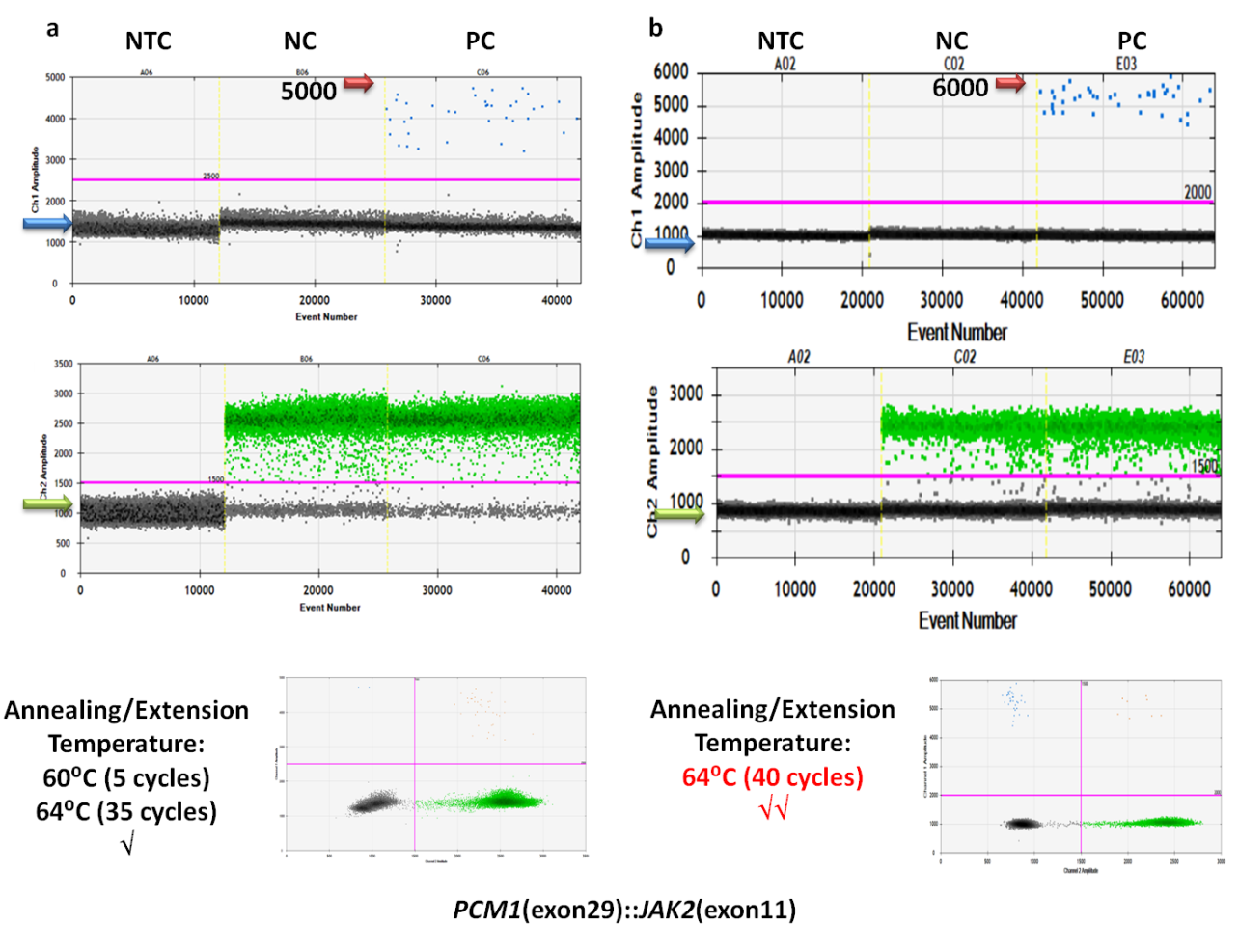


**Supplementary Figure S2**: Optimization of *PCM1*(exon 29)::*JAK2*(exon 11) ddPCR assay. 1-D and 2-D plots of *PCM1*(exon 29)::*JAK2*(exon 11) ddPCR assay performed at 60 and 64°C (a) and 64°C only (b) for annealing/extension step. Higher annealing/extension temperature enhanced the PCR efficiency as demonstrated by the more compact FAM-positive cluster (red arrow). The background is also cleaner as shown by the tighter FAM- (blue arrow) and HEX-negative clusters (green arrow). (NTC: no template control, NC: negative control, PC: positive control)


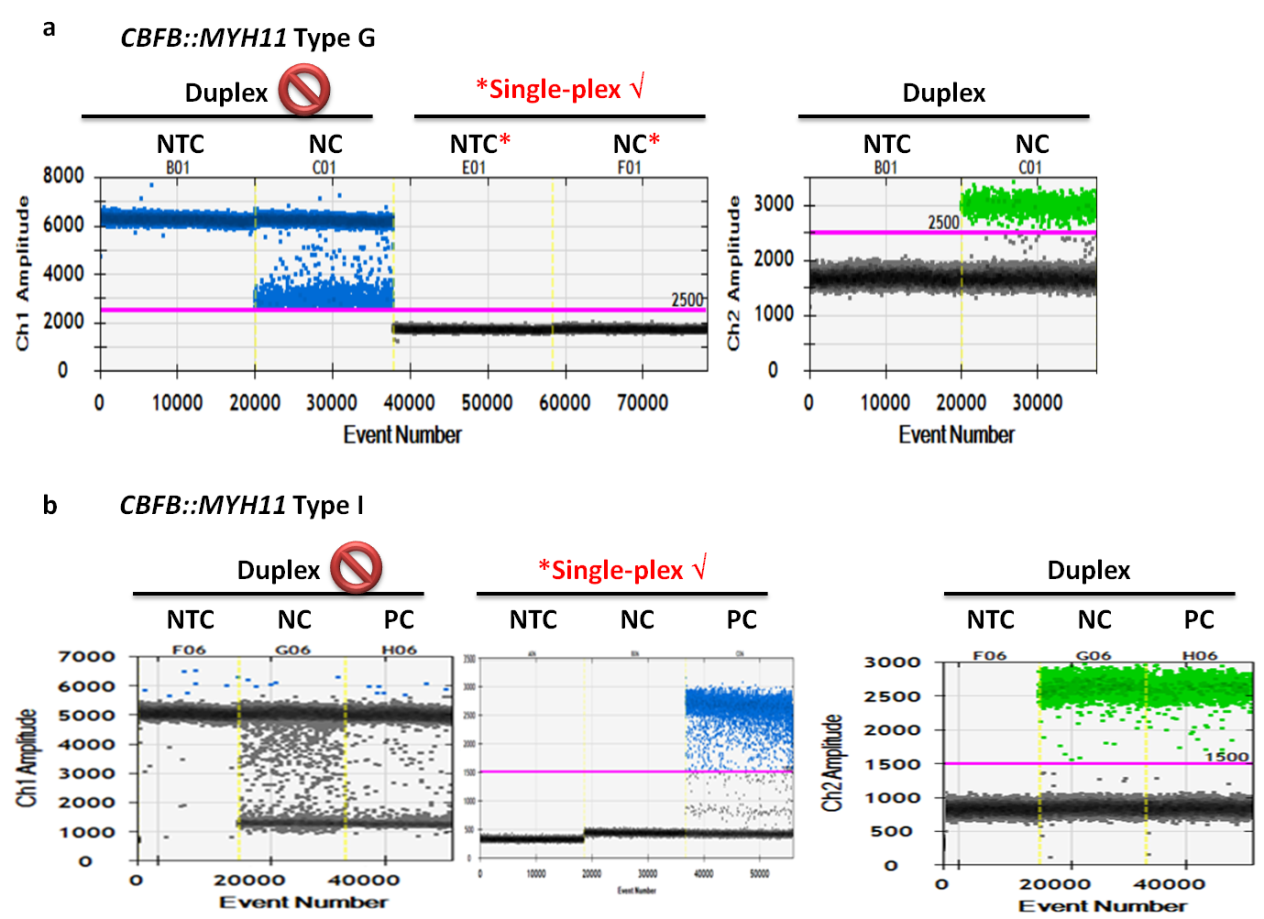


**Supplementary Figure S3**: Optimization of *CBFB*::*MYH11* ddPCR assays. 1-D plots of *CBFB::MYH11* Type G (a) and Type I (b) ddPCR assays performed in duplex with *ABL1*, causing an abnormal increase in fluorescent signal intensity of the negative droplets in the FAM channel. Once the ddPCR assays for target and reference genes were performed in discrete wells (i.e. single-plex), the positive and negative droplets in the FAM channel were separated well. The *ABL1* assay in the HEX channel was not affected even when performed in duplex. (NTC: no template control, NC: negative control, PC: positive control)


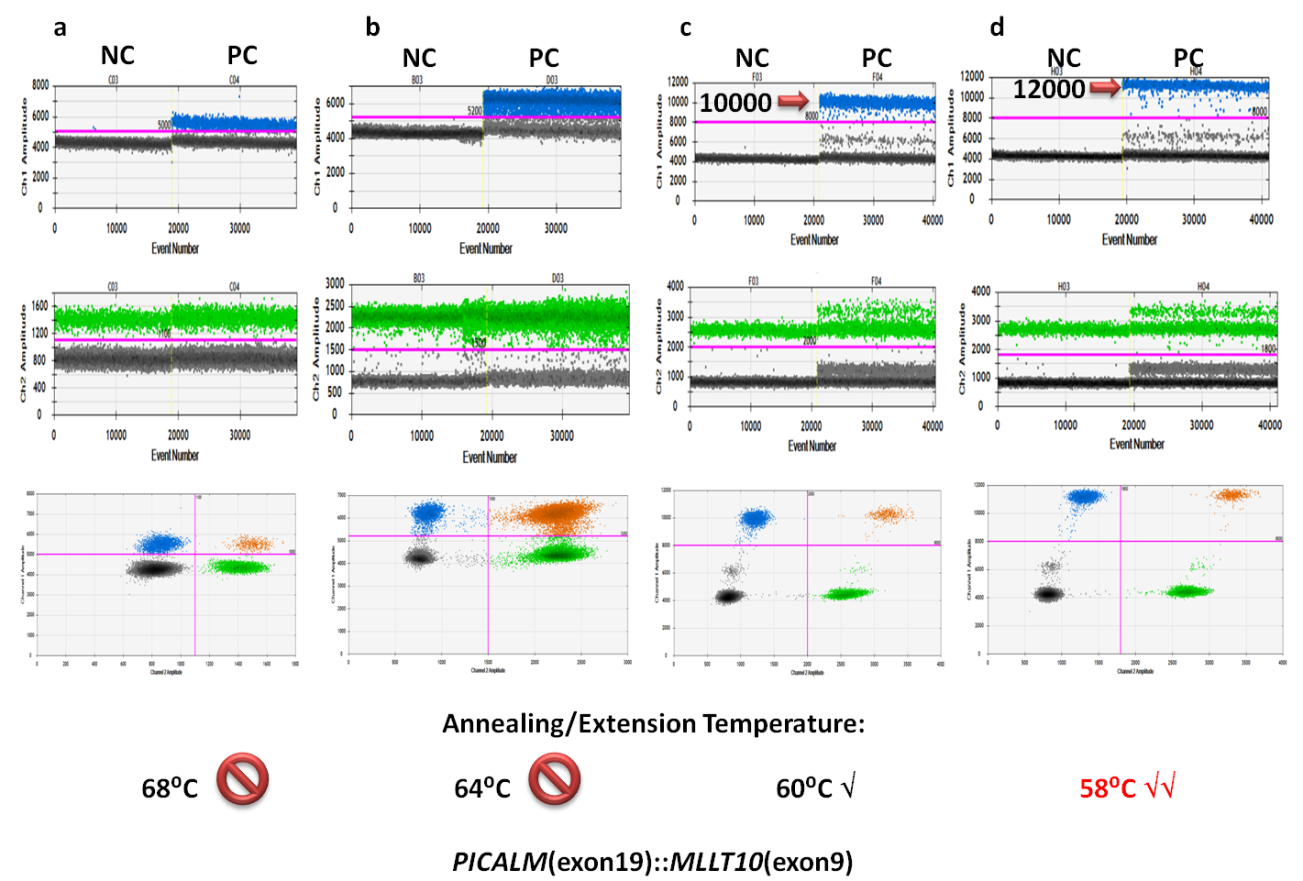


**Supplementary Figure S4**: Optimization of *PICALM*(exon 19)::*MLLT10*(exon 9) ddPCR assay. 1-D and 2-D plots of *PICALM*(exon 19)::*MLLT10*(exon 9) ddPCR assay performed at various annealing/extension temperature, from 68 down to 58°C (a-d). Lower annealing/extension temperature enhanced the PCR efficiency as demonstrated by the more compact FAM-positive cluster with higher fluorescent signal intensity (red arrow). (NC: negative control, PC: positive control)


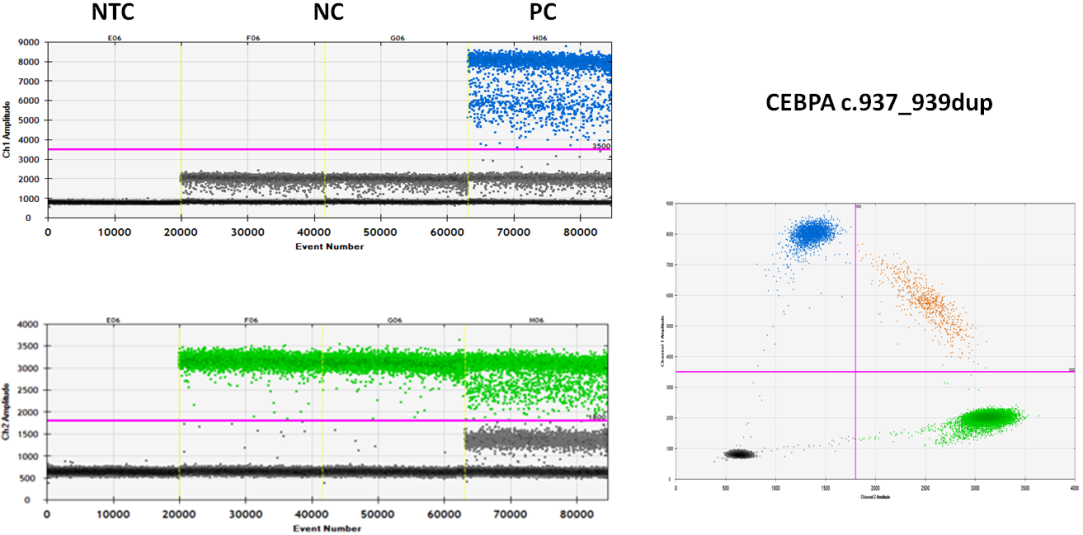


**Supplementary Figure S5**: Optimization of CEBPA c.937_939dup ddPCR assay. 1-D and 2-D plots of CEBPA c.937_939dup ddPCR assay. Good separation of positive and negative clusters in both channels. (NTC: no template control, NC: negative control, PC: positive control)


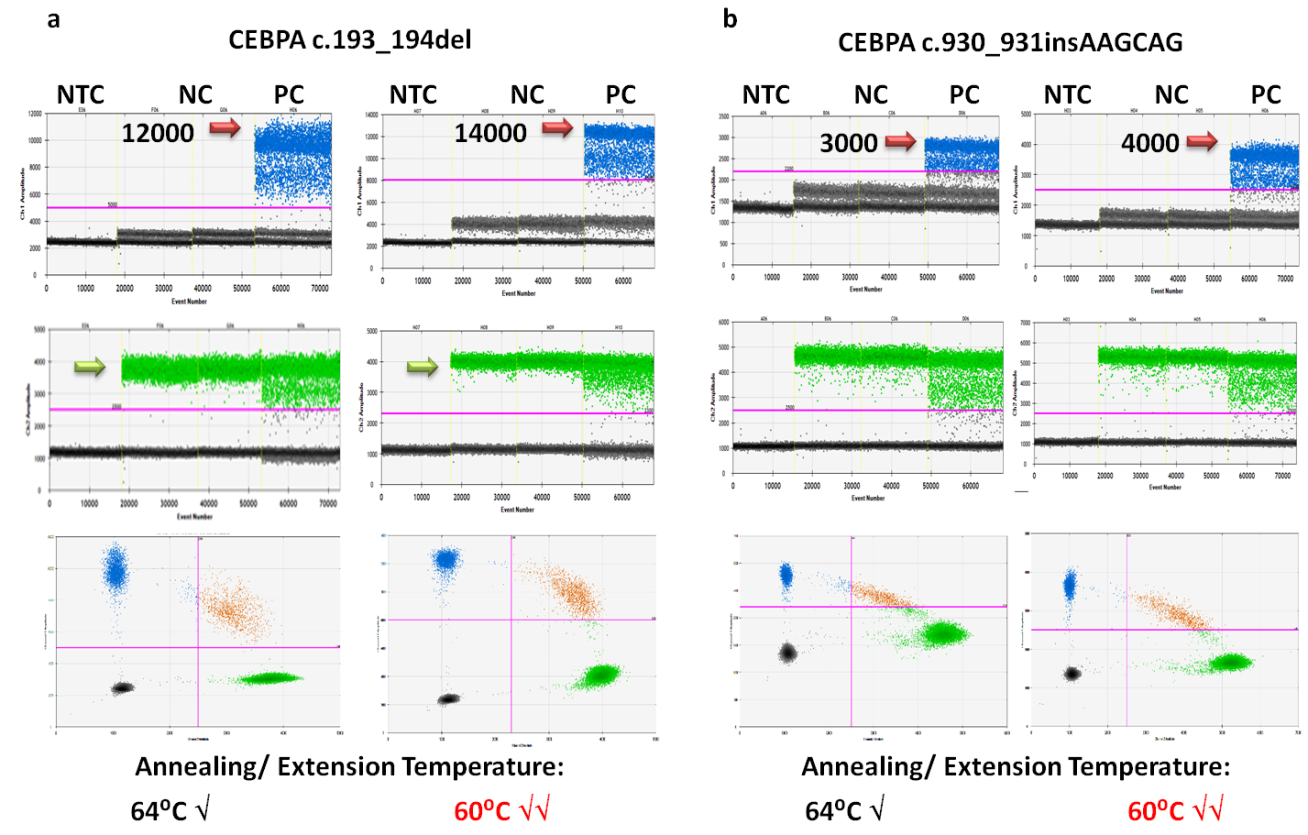


**Supplementary Figure S6**: Optimization of CEBPA c.193_194del and c.930_931insAAGCAG ddPCR assays. 1-D and 2-D plots of CEBPA c.193_194del (a) and c.930_931insAAGCAG (b) ddPCR assays performed at 64 and 60°C for annealing/extension step. Lower annealing/extension temperature enhanced the PCR efficiency as demonstrated by the higher fluorescent signal intensity in the FAM-positive droplets (red arrow). The HEX-positive cluster was also more compact (green arrow, a). (NTC: no template control, NC: negative control, PC: positive control)


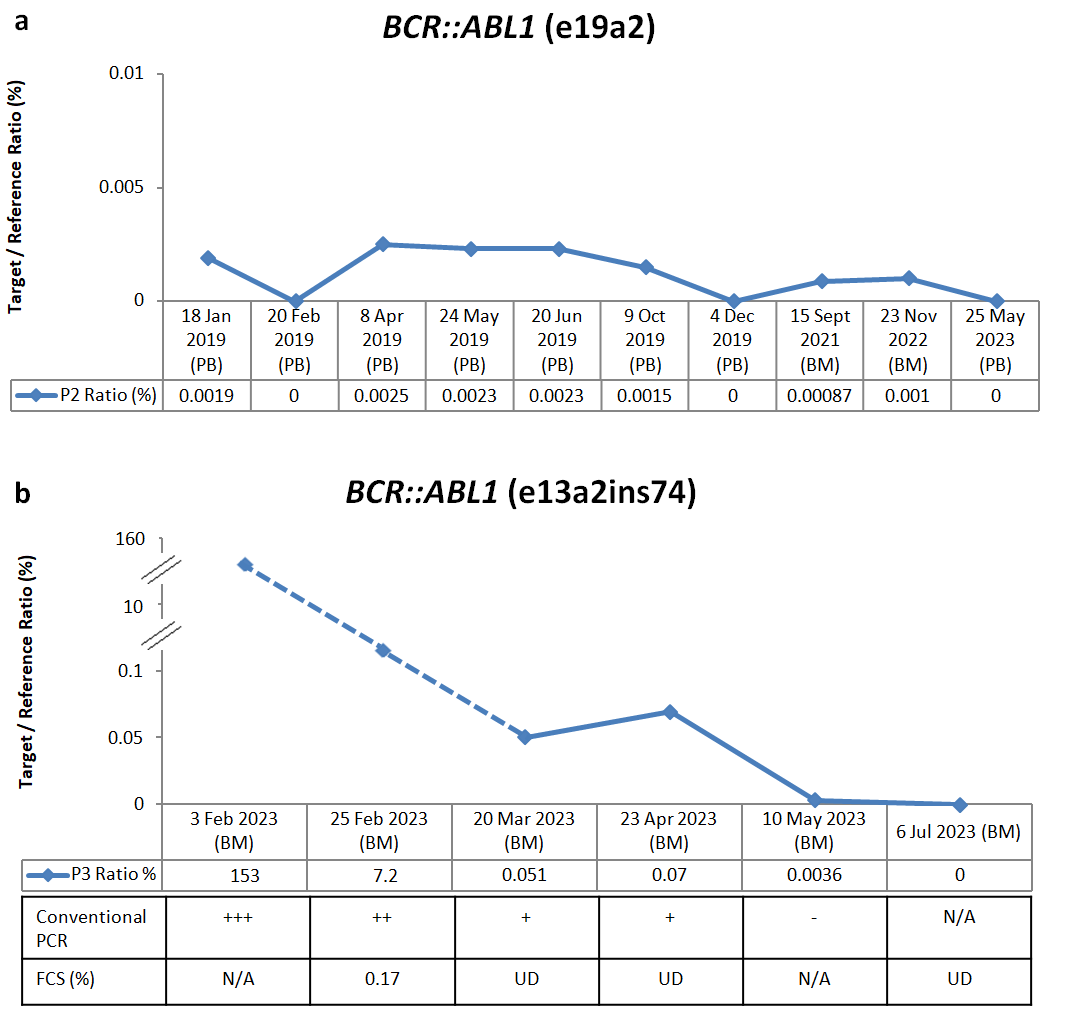


**Supplementary Figure S7**: MRD monitoring for Patient 2 and 3. (a) Serial MRD monitoring for *BCR::ABL1* (e19a2) by ddPCR. (b) Serial MRD monitoring for *BCR::ABL1* (e13a2ins74) by ddPCR, conventional PCR and multiparametric flow cytometry (FCS). (BM: bone marrow, +++: strongly positive, ++: positive, + weakly positive, -: negative, UD: undetectable, N/A: not applicable)


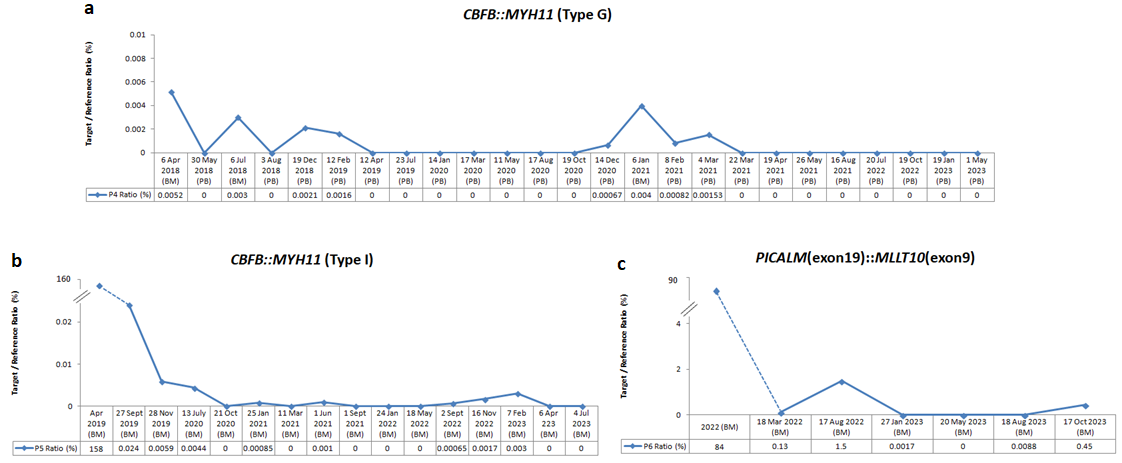


**Supplementary Figure S8**: MRD monitoring for Patient 4-6. Serial MRD monitoring for *CBFB::MYH11* Type G (a), Type I (b) and *PICALM*(exon19)::*MLLT10*(exon9) (c) by ddPCR. (BM: bone marrow, PB: peripheral blood)


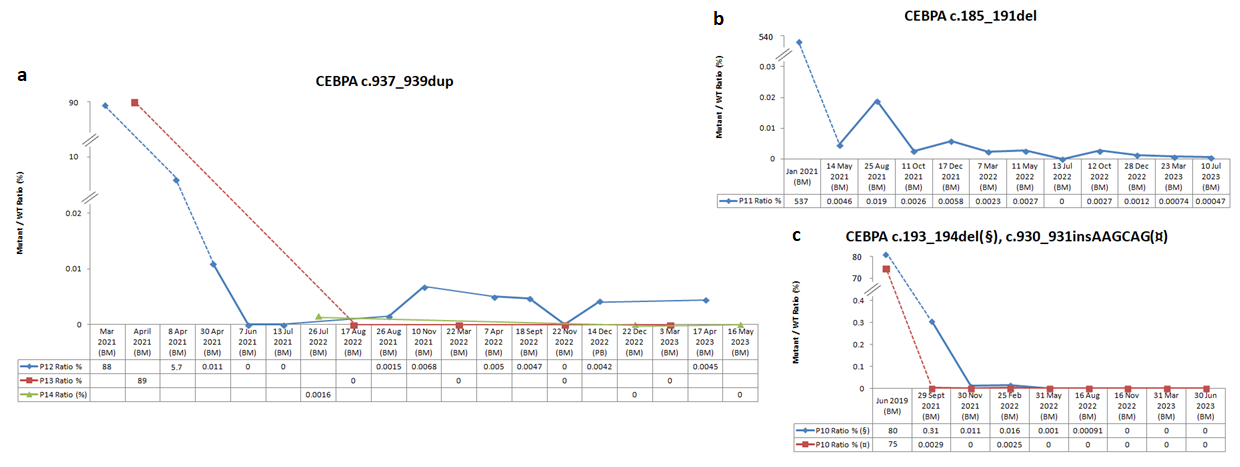


**Supplementary Figure S9**: MRD monitoring for Patient 10-14. Serial MRD monitoring for CEBPA c.937_939dup (a), c.185_191del (b), c.193_194del and c.930_931insAAGCAG (c) by ddPCR. (BM: bone marrow)


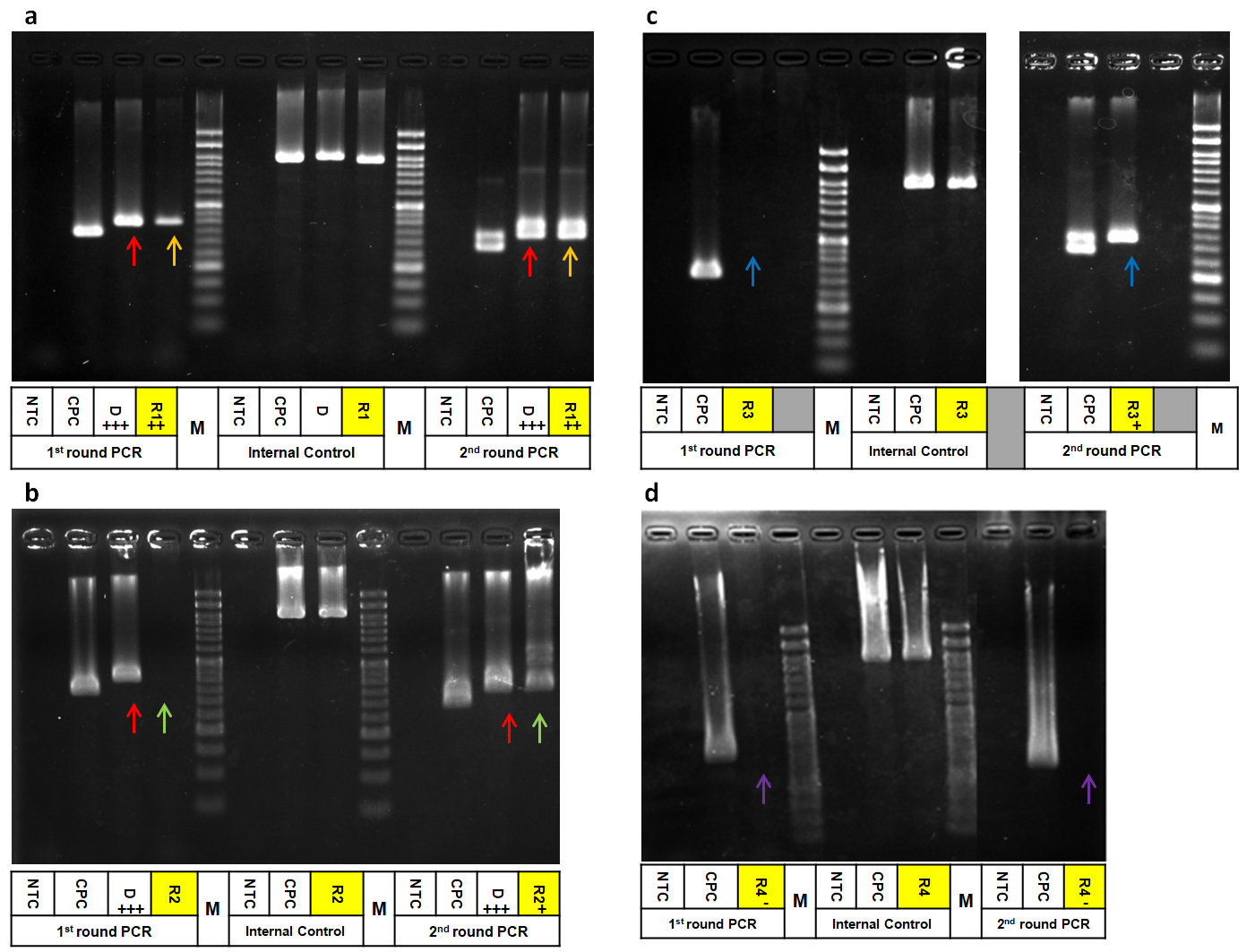


**Supplementary Figure S10**: MRD monitoring for Patient 3 by conventional PCR. Serial MRD monitoring for *BCR::ABL1* (e13a2ins73) on (a) the diagnostic sample collected on 3 Feb 2023 (D, red arrow) and the first reassessment sample (R1, orange arrow) collected on 25 Feb 2023, (b) the second reassessment sample (R2, green arrow) collected on 20 Mar 2023, (c) the third reassessment sample (R3, blue arrow) collected on 23 Apr 2023 and (d) the fourth reassessment sample (R4, purple arrow) collected on 10 May 2023 by conventional PCR. (NTC: no template control, CPC: commercial positive control of *BCR::ABL1* p210 b2a2, D: diagnostic sample, R: reassessment samples, +++: strongly positive, ++: positive, + weakly positive, -: negative, M: 50 bp DNA ladder)

|  | Dilution Series (%) | Linear Range (Copies / 20 uL) |
| --- | --- | --- |
| *ABL1* | 1 – 100 | 143 – 14,610 |
| *BCR::ABL1* (e19a2) | 0.5 – 100 | 7 – 1,284 |
| *BCR::ABL1* (e23a2ins52) | 0.5 – 100 | 11 – 2,740 |
| *BCR::ABL1* (e13a2ins74) | 0.025 – 100 | 7 – 33,820 |
| *CBFB::MYH11* (Type G) | 0.01 – 100 | 10 – 79,900 |
| *CBFB::MYH11* (Type I) | 0.05 – 100 | 8 – 14,720 |
| *PCM1*(exon29)::*JAK2*(exon11) | 0.01 – 100 | 5 – 48,940 |
| *KMT2A*(exon8)::*ELL*(exon2) | 0.1 – 100 | 6 – 6,590 |
| *PICALM*(exon19)::*MLLT10*(exon9) | 0.025 - 100 | 7 – 3,180 |

**Supplementary Table S1**: Analytical performance evaluation for RNA-based ddPCR assays. The dilution series in % and linear range in copies/20 uL used for the linearity assessment and regression analysis of the reference gene (*ABL1*) and various atypical fusion transcripts ddPCR assays.

|  | Mutant Allele | | Wild-Type Allele | |  |
| --- | --- | --- | --- | --- | --- |
|  | Dilution Series (%) | Linear Range (Copies / 20 uL) | Dilution Series (%) | Linear Range (Copies / 20 uL) | NGS vs. ddPCR VAF (Ratio) |
| CEBPA c.193_194del | 0.125 – 100 | 8 – 6,340 | 0.125 – 100 | 13 – 7,960 | 0.45 vs. 0.44 |
| CEBPA c.930_931insAAGCAG | 0.125 – 100 | 7 – 5,500 | 0.125 – 100 | 10 – 7,360 | 0.42 vs. 0.43 |
| CEBPA c.185_191del | 0.25 – 100 | 15 – 7,550 | 0.5 – 100 | 8 – 1,406 | 0.84 vs. 0.84 |
| CEBPA c.937_939dup | 0.25 – 100 | 9 – 4,380 | 0.25 – 100 | 14 – 4,960 | 0.44 vs. 0.47  0.47 vs. 0.47 |

**Supplementary Table S2**: Analytical performance evaluation for DNA-based ddPCR assays. The dilution series in % and linear range in copies/20 uL used for the linearity assessment and regression analysis of the CEBPA mutations ddPCR assays. The variant allele frequencies (VAF) in ratio of the patients’ diagnostic samples obtained by ddPCR were comparable to the pre-defined VAF by NGS (±0.5LOG).

| Primer / Probe Name | Primer / Probe Sequence |
| --- | --- |
| 1. ddABL1_9F | 5’-CACCAAGACGAG GACCTCCAGGA-3’ |
| 1. ddABL1_10R | 5’-AAGGCGCTCATCTTCATTCAGGC |
| 1. Probe_ABL | 5’-HEX/GAGCGATCCTCTGGACCATGAG/3’Iowa Black FQ |
| 1. p230_BCRex19F | 5’-GCATGGAGGAGGTGGGCATCTAC-3’ |
| 1. p230_ABLex2R | 5’-GGTTGGGGTCATTTTCACTGGGTC-3’ |
| 1. Probe_p230 | 5’-6-FAM/CTTCGACGTCAAAGCCCTTCAGC/3’Iowa Black FQ |
| 1. BCR_ABL_DelIns52_F | 5’-AGCCTATCACCATGACTGACAGCTG-3’ |
| 1. BCR_ABL_DelIns52_R | 5’-CAGATGCTACTGGCCGCTGAAG-3’ |
| 1. Probe_BCR_ABL_delins52 | 5’-6-FAM/CTGCAGCTGGAGTGTTGGGATTACA/3’Iowa Black FQ |
| 1. BCR-E13F1 | 5’-CAGATGCTGACCAACTCGTGTGTGA-3’ |
| 1. ABL1-E2R1 | 5’-GAGCGGCTTCACTCAGACCCTGA-3’ |
| 1. BCR-ABL1e13a2ins74 Probe1 | 5’-6-FAM/CAGACTGTCCACAGCATAACCCACCT/3’Iowa Black FQ |
| 1. ddCBFB_4F2 | 5’-AGGCTCCCATGATTCTGAATGGAGT-3’ |
| 1. ddMYH11_30R2 | 5’-AGCTGGGAACTGAGGGACGC-3’ |
| 1. Probe_CBFB_MYH11 | 5’-6-FAM/GAGCGAGCCCAGAATGAAGTTGA/3’Iowa Black FQ |
| 1. Kadkol-INV16-1 [10] | 5’-GCAGGGAGAACAGCGACAAACA-3’ |
| 1. Kadkol-INV16-2 [10] | 5’-TCTGGAGGCACGGGCATC-3’ |
| 1. CBFB4MYH11x34_Probe | 5’-6-FAM/GCGAGCCCAGCTTCACGAGT/3’Iowa Black FQ |
| 1. PCM1_ex29F | 5’-GAATTGATACTCAGCAGCTGGAC-3’ |
| 1. JAK2_ex11R | 5’-TTGTCCCACTGAGGTTGTACTCTT-3’ |
| 1. Probe_PCM1_JAK2 | 5’-6-FAM/TCCTTTTTTGAAGCGAGAAAATGTCA/3’Iowa Black FQ |
| 1. KMT2Ax7_F | 5’-CAGAATCAGGTCCAGAGCAGAGCAA-3’ |
| 1. ELLx3_R | 5’-GATGTGCCCTTGGCTTCCTTGAA-3’ |
| 1. KMT2Ax8_ELLx2_MutProbe | 5’-6-FAM/AGGATTCTGTTTCACTGAGGCCATC/3’Iowa Black FQ |
| 1. ddPICALMex19F | 5’-GTTCCTGTAATGACGCAACCAACCT-3’ |
| 1. ddMLLT10ex9R | 5’-GGTGATGGTTCTGGCTGCTTCTTG-3’ |
| 1. ddPICALM_MLLT10_Probe19_9 | 5’-6-FAM/AGGAGCACAGAAATATAAAGAGAAGGA/3’Iowa Black FQ |

**Supplementary Table S3**: List of primers and probes for RNA-based ddPCR assays. The primers and probes sequences were listed from 5’ to 3’ direction. (FQ: fluorescence quencher)

| Primer / Probe Name | Primer / Probe Sequence |
| --- | --- |
| 1. ddCEBPAc185DelF | 5’-CCACCTGCCGCCCCGGAGCCGC-3’ |
| 1. ddCEBPAc185DelR | 5’-GCCAGGAACTCGTCGTTGAAGG-3’ |
| 1. ddCEBPAc193_194WT_Probe | 5’-HEX/CATCGACATCAGCGCCTACATCG/3’Iowa Black FQ |
| 1. ddCEBPAc193_194del_Probe | 5’-6-FAM/ CCATCGACATCCGCCTACATCG/3’Iowa Black FQ |
| 1. ddCEBPAc930_931F | 5’-GACAAGAACAGCAACGAGTACCG-3’ |
| 1. ddCEBPAc930_931R1 | 5’-AGTTCGCGGCTCAGCTGTT-3’ |
| 1. ddCEBPAc930_931WT_Probe | 5’-HEX/ACGTGGAGACGCAGCAGAAG/3’Iowa Black FQ |
| 1. ddCEBPAc930_931insAAGCAG_Probe | 5’-6-FAM/GGAGACGAAGCAGCAGCAGA/3’Iowa Black FQ |
| 1. CEBPAc185Del_WTProbe | 5’-HEX/GAGACGTCCATCGACATCAGCGCC/3’Iowa Black FQ |
| 1. CEBPAc185Del_MutProbe | 5’-6-FAM/GAGACGTCCACAGCGCCTACATCG/3’Iowa Black FQ |
| 1. CEBPA_c937DupF | 5’-TGGACAAGAACAGCAACGAG-3’ |
| 1. CEBPA_c937DupR | 5’-CGGTCATTGTCACTGGTCA-3’ |
| 1. CEBPA_c937_939WT_Probe | 5’-HEX/GGAGACGCAGCAGAAGGTGCTGGAGC/3’Iowa Black FQ |
| 1. CEBPA_c937_939dup_Probe | 5’-6-FAM/AGACGCAGCAGAAGAAGGTGCTGGAGC/3’Iowa Black FQ |

**Supplementary Table S4**: List of primers and probes for DNA-based ddPCR assays. The primers and probes sequences were listed from 5’ to 3’ direction. (FQ: fluorescence quencher)

| Cycling Step | Temperature (⁰C) | Time | Number of Cycles | Ramp Rate |
| --- | --- | --- | --- | --- |
| Restriction Enzyme Digestion (*) | 37 | 1 hour | 1 | 2⁰C per sec |
| Enzyme Activation | 95 | 10 min | 1 |  |
| Denaturation 1 | Variable (a) | 30 sec | 5 |  |
| Annealing/Extension 1 | Variable (b) | Variable (d) |  |  |
| Extension 1 (#) | 72 | 2 min |  |  |
| Denaturation 2 | Variable (a) | 30 sec | 35 |  |
| Annealing/Extension 2 | Variable (c) | Variable (e) |  |  |
| Extension 2 (#) | 72 | 2 min |  |  |
| Enzyme Deactivation | 95 | 10 min | 1 |  |
| Droplet Stabilization | 4 | 30 min | 1 |  |
| Hold | 4 | indefinitely | - |  |

(*) only required in Mutation (DNA) assays

(#) only required in *CBFB::MYH11* Type I assay

**Supplementary Table S5**: Thermal cycling conditions for all listed ddPCR assays. Variable (a) to (e) indicated the conditions subjecting to changes during optimization.

| RNA / DNA | Assay  (Duplex (D) or Single-plex (S)) | Primer Pairs and Probes  (Refer to Supplementary Table S3 and S4) | Thermal Cycling Conditions  (Refer to Refer to Supplementary Table S5) |
| --- | --- | --- | --- |
| Fusion Transcript Expression (RNA) | *BCR::ABL1* (e19a2) / *ABL1* (D) | 1, 2, 3; 4, 5, 6 | a = 94, b = 60, c = 64,  d & e = 1 min |
|  | *BCR::ABL1* (e23a2ins52) / *ABL1* (D) | 1, 2, 3; 7, 8, 9 |  |
|  | *BCR::ABL1* (e13a2ins74) / *ABL1* (D) | 1, 2, 3; 10, 11, 12 |  |
|  | *CBFB::MYH11* (Type G) / *ABL1* (S) | 1, 2, 3; 13, 14, 15 |  |
|  | *KMT2A*(exon8)::*ELL*(exon2) / *ABL1* (S) | 1, 2, 3; 22, 23, 24 |  |
|  | *CBFB::MYH11* (Type I) / *ABL1* (S) | 1, 2, 3; 16, 17, 18 | a = 94, b & C = 62,  d & e = 1 min 30 sec |
|  | *PCM1*(exon29)::*JAK2*(exon11) / *ABL1* (D) | 1, 2, 3; 19, 20, 21 | a = 94, b & c = 64,  d & e = 1 min |
|  | *PICALM*(exon19)::*MLLT10*(exon9) / *ABL1* (D) | 1, 2, 3, 25, 26, 27 | a = 94, b & c = 58,  d & e = 1 min |
| Mutation  (DNA) | CEBPA c.193_194del / Wild-type (D) | 28, 29, 30, 31 | a = 96, b & c = 60,  d & e = 1 min 30 sec |
|  | CEBPA c.930_931insAAGCAG / Wild-type (D) | 32, 33, 34, 35 |  |
|  | CEBPA c.185_191del / Wild-type (D) | 28, 29, 36, 37 | a = 96, b & c = 64,  d & e = 1 min 30 sec |
|  | CEBPA c.937_939dup / Wild-type (D) | 38, 39, 40, 41 | a = 94, b = 60, c = 64,  d & e = 1 min 30 sec |

**Supplementary Table S6**: The set up of ddPCR assays for fusion transcripts expression and mutation detection.
